# Enhanced streamflow forecasting using hybrid modelling integrating glacio-hydrological outputs, deep learning and wavelet transformation

**DOI:** 10.1038/s41598-025-87187-1

**Published:** 2025-01-22

**Authors:** Jamal Hassan Ougahi, John S Rowan

**Affiliations:** 1 UNESCO Centre of Water Law, Policy & Science, University of Dundee, Dundee, UK; 2 Higher Education Department, Government of the Punjab, Lahore, Pakistan

**Keywords:** Artificial Intelligence (AI), Hydrological modelling, Hybrid models, Machine learning, Deep learning, Glacier-runoff simulation, Upper Indus Basin, Hindu-Kush Karakorum Himalaya region, Hydrology, Cryospheric science

## Abstract

Understanding snow and ice melt dynamics is vital for flood risk assessment and effective water resource management in populated river basins sourced in inaccessible high-mountains. This study provides an AI-enabled hybrid approach integrating glacio-hydrological model outputs (GSM-SOCONT), with different machine learning and deep learning techniques framed as alternative ‘computational scenarios, leveraging both physical processes and data-driven insights for enhanced predictive capabilities. The standalone deep learning model (CNN-LSTM), relying solely on meteorological data, outperformed its counterpart machine learning and glacio-hydrological model equivalents. Hybrid models (CNN-LSTM1 to CNN-LSTM15) were trained using meteorological data augmented with glacio-hydrological model outputs representing ice and snow-melt contributions to streamflow. The hybrid model (CNN-LSTM14), using only glacier-derived features, performed best with high NSE (0.86), KGE (0.80), and R (0.93) values during calibration, and the highest NSE (0.83), KGE (0.88), R (0.91), and lowest RMSE (892) and MAE (544) during validation. Finally, a multi-scale analysis using different feature permutations was explored using wavelet transformation theory, integrating these into the final hybrid model (CNN-LSTM19), which significantly enhances predictive accuracy, particularly for high-flow events, as evidenced by improved NSE (from 0.83 to 0.97) and reduced RMSE (from 892 to 442) during validation. The comparative analysis illustrates how AI-enhanced hydrological models improve the accuracy of runoff forecasting and provide more reliable and actionable insights for managing water resources and mitigating flood risks - despite the paucity of direct measurements.

**Supplementary Information:**

The online version contains supplementary material available at 10.1038/s41598-025-87187-1.

## Introduction

Floods are a major natural disaster that can cause significant socioeconomic and human losses worldwide^1^. The Centre for Research on the Epidemiology of Disasters reports that whilst flooding ranked second in terms of the number of people affected (33%) after storms (35%), it was responsible for the highest percentage of deaths, about 43.5% globally^2^. This highlights the critical importance of accurate river and flood forecasting in water resource management. In the summer of 2022, severe meteorological disasters, including heavy monsoon rainfall and glacial melt, led to widespread flooding in South Asia, particularly affecting Pakistan, Sri Lanka, Afghanistan, Bangladesh, and India^3^. In Pakistan, floods resulted in nearly 1,700 deaths, over 12,900 injuries, and damage to more than 1.3 million homes, displacing about 7.9 million people^4^. Therefore, effective forecasting is essential to make informed decisions and policies for monitoring and mitigating flood damage. However, predicting streamflow can be challenging due to issues such as the paucity reliable long-term hydrological data, limited capacity in relevant scientific expertise and disconnects with decision-makers in the policy community.

Hydrologic models have evolved significantly since the 1850s, becoming powerful tools for forecasting floods, droughts, and managing water resources^5^. Hydrological models are widely used to understand the water cycle and evaluate the potential impacts of water management policies, water resource management effectively, predicting water availability, flood control and overall sustainable development^6–8^. Physical or process-based hydrological models which represent catchment hydrological processes through mathematical equations are commonly used to simulate streamflow^9^. Initially driven by advances in numerical mathematics and computing, modern hydrologic models now integrate complex Earth systems, including climate, weather, atmospheric circulation, and geospatial data^10,11^. In recent decades, process-based hydrological modelling has advanced due to advancements in our understanding of hydrological processes, the availability of global gridded datasets^12^, and increased computing power^13^. Numerous process-based hydrological models have been developed and are extensively used to analyse streamflow dynamics and the interactions between land use, climate, and agriculture^14,15^. These models require high-resolution, long-term hydrometeorological data for proper calibration and validation^16^. However, in glacier-melt-dominated basins, process-based hydrological models encounter significant challenges due to the high uncertainty in input data^17,18^. Improving the prediction capability of hydrological models could enhance water resource management in data scarce mountainous regions. In addition to process-based hydrological models, machine learning methods should be explored to achieve more accurate and reliable streamflow simulations in data-scarce river basins.

Global warming has significantly increased slope instability in high-elevation areas, leading to increased rockfalls and debris flows during spring and summer months^19^. Seasonal and perennial snowpacks and glaciers serve as cold water reservoirs, providing runoff during the late spring, summer, early autumn and steady baseflow round the year^20,21^. Besides supplying runoff, snow and ice are also vital in regulating climate, and their role in ecosystem services and the cooling effect critical to the surface energy budget^22^. The Upper Indus Basin (UIB) located in the Hindu-Kush Karakoram Himalaya (HKH) region is highly glacierized and heavily reliant on snow and glacier melt for its river flows^23–27^. Over half of its annual flow originates from snow and glacier fed sub basins like the Hunza, Gilgit, Shyok and Astore River basins^28^. In this semi-arid basin, careful management of water resources is crucial for sustaining livelihoods, ensuring food security, generating hydroelectric power, and supporting the economic development of millions of people living downstream of the UIB^29,30^.

In the UIB, warming is affecting the hydrology due to accelerated glacial melting and shifting snowmelt from summer to spring^31,32^. Since snowmelt and glacier melt, which contribute significantly to streamflow, it is essential to integrate these processes into traditional hydrological models^33^. Accurately simulating the timing and contribution of snow and glacier melt is essential for ensuring a sustainable year-round water supply for irrigation and hydroelectric power generation, and flood risk assessment^34,35^. However, developing a reliable hydrological model to accurately simulate runoff contribution from snow and glacier ice melt in particular remains a challenge for effective water resource management, particularly in the context of rising global temperatures^36^.

### Modelling options

To understand the proportion of total runoff attributable to various processes and to make informed predictions about future water availability, glacio-hydrological models are essential^37^. The well-known Glacier-Snow Melt Soil CONTribution model (GSM-SOCONT)^38^ has been employed in different mountainous and glacierized basins around the world^23,39–42^ and for the first time in the UIB. The GSM-SOCONT model can be applied in either a lumped or semi-distributed manner. The key feature of this model is its ability to separate and simulate different components of runoff like snowmelt, glacial melt, baseflow, surface runoff, and soil infiltration. This makes it a powerful tool for understanding and predicting water flow in regions influenced by snow and glaciers.

Despite considerable advancements in hydrological modelling, uncertainties persist due to factors such as input and calibration data, model structure, and parameters^43^. These uncertainties continue to pose a significant challenge in accurately simulating hydrological systems. In response, researchers widely use data-driven models which evolved from traditional statistical analysis based on mathematical regression and have progressed towards incorporating advanced computational intelligence techniques^44,45^. In the UIB, machine learning (ML) and artificial intelligence (AI) techniques have been increasingly employed to improve the accuracy and efficiency of hybrid models for streamflow forecasting^46,47^ and elsewhere^48–50^.

Data-driven approaches, which focus on optimizing model parameters to improve prediction accuracy, often outperform traditional physically based models^51,52^. These approaches effectively capture complex data patterns and improve predictions^53^. While ML models provide fast predictions and can perform well with extensive historical data, they face challenges with data needs, spatial variability, and extreme flows^9^. Despite their limitations, these models are valuable when key processes are well-represented^54,55^. Deep learning (DL) models have gained significant attention due to their ability to handle complex tasks without requiring domain specific knowledge. Convolutional Neural Networks (CNNs) are widely used in contrast to multilayer perceptron (MLP) and recurrent neural networks (RNNs) due to fewer parameters. CNNs are often used in combination with other models, such as Long Short-Term Memory (LSTM) networks, to create hybrid models (e.g., CNN-LSTM)^56,57^. The Hybrid CNN-LSTM model has good prediction accuracy in streamflow forecasting^58^. ML and deep learning models, particularly in hybrid forms, have shown promise in enhancing streamflow forecasts, especially in regions where process-based models are less effective^53,59^.

In the UIB, data scarcity complicates the accurate quantification of glacial melt contributions to streamflow and introduces uncertainties in glacio-hydrological models. This lack of data hinders the representation of hydrological processes, understanding of water availability, and validation of model outputs^60^. The accuracy of these models is crucial for effective water resource management. For example, Garee et al.^61^ successfully calibrated the SWAT model to simulate streamflow but overlooked the contribution of glacial melt in the highly glacierized sub-basins of the UIB. Lateef et al.^28^ compared the SPHY and SRM models to assess their ability to simulate the contributions of glacier melt, snowmelt, and rainfall in the UIB. Nazeer et al.^62^ used global precipitation data and a simplified precipitation-runoff model to simulate snow and glacier melt in the Gilgit Basin. These studies either ignored glacier melt or used inadequate validation methods, resulting in gaps in understanding and accurately simulating streamflow components in the UIB.

A review of over 145 glacio-hydrological modelling studies worldwide suggests that modelers can achieve seemingly good calibration results by adjusting the parameters of snow and ice accumulation and melt processes, though often for wrong reasons^17^. This leads to compensation errors in highly glacierized basins, obscuring the true uncertainties in the input data. Therefore, it is essential to validate ice melt contributions in hydrological models using alternative data sources especially in the UIB. In this study, glacier parameters in the GSM-SOCONT model are refined by integrating monthly glacier runoff estimates from the Python Glacier Evolution Model (PyGEM)^63^, allowing for improved calibration of glacier-specific parameters, even in the absence of direct glacier discharge observations. This approach helps to refine the degree-day factors which play a critical role for accurately simulating glacier melt, a parameter often overlooked or inadequately calibrated in traditional hydrological models due to limited data. This study aims to develop a hybrid modelling approach by integrating a glacio-hydrological model, ML techniques and wavelet theory to address current gaps and improve hydrological simulation accuracy in the region.

## Methodology

### Study site

**Fig. 1:**
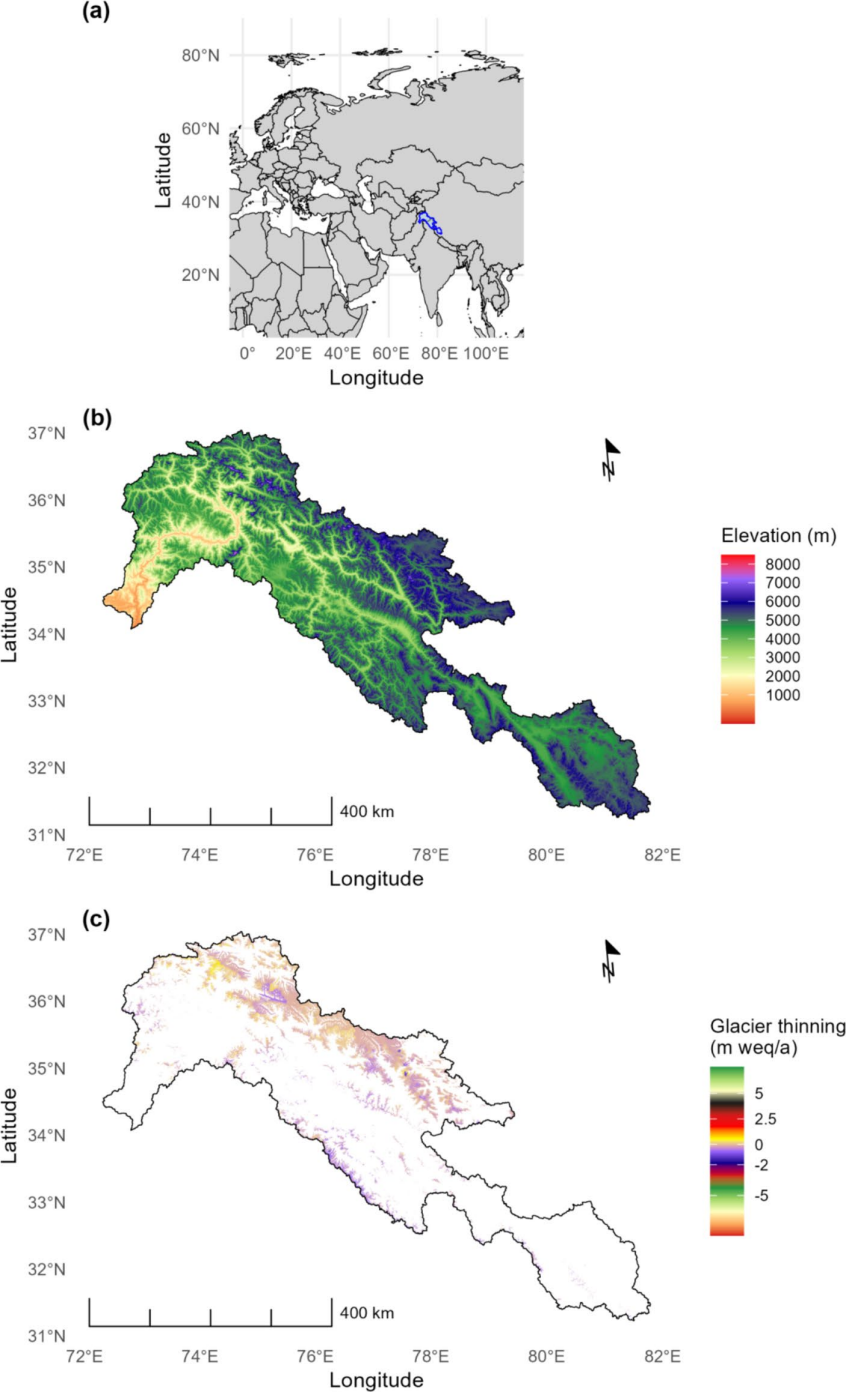
Map showing: (**a**) the location of the Upper Indus Basin on a world map, (**b**) glacier thinning data within the UIB, and (**c**) the elevation map of the Upper Indus Basin.

The Indus River, one of southern Asia’s largest rivers, is 2,880 km long with a drainage area of 912,000 km^2^, covering parts of Pakistan, India, China, and Afghanistan^64^. The Upper Indus Basin (UIB) is situated in the north of Pakistan, extending across northern India and a portion of western China, with coordinates from 31.0° to 37.0° N latitude and 72.0° E to 82.0° E longitude (Fig. 1). The UIB with about 90% of its area located between the Himalaya and Karakoram ranges plays a crucial in agriculture and power generation of Pakistan^65^. The Indus River derives 60–70% of its water from seasonal precipitation and snowmelt in the Himalaya-Karakoram-Hindukush (HKH) region^66,67^. The Indus Basin irrigation network is one of the largest in the world, irrigating 17 million hectares of cultivable land^68^ and contributes to 90% of the agricultural output^69^.

The climate of the UIB is shaped by a complex interaction between monsoon circulation, westerlies, and the topography of the region^70,71^. This region relies heavily on snowfall for precipitation with monsoon has a stronger influence along the Himalayan arc while the mid-latitude westerlies become more dominant at the junction of the Karakoram, Pamir, and Hindu-Kush Mountain ranges^67^. Maximum temperatures had increased during winter, spring, and autumn, while showing a decline in summer, indicating a cooling trend during the summer months^31^. Any changes in precipitation patterns, the timing of seasonal shifts, or the retreat and thinning of glaciers could significantly disrupt water availability for irrigation, leading to adverse effects on agriculture and livelihoods^72^.

### Datasets

ERA5 is the fifth generation ECMWF (European Centre for Medium-Range Weather Forecasts) reanalysis product providing global climate and weather data spanning the past eight decades^73^. ERA5 offers notable advancements over ERA-Interim, including improved spatial and temporal resolution with hourly estimates of atmospheric variables at a 31 km horizontal resolution and 137 vertical layers.

**Fig. 2:**
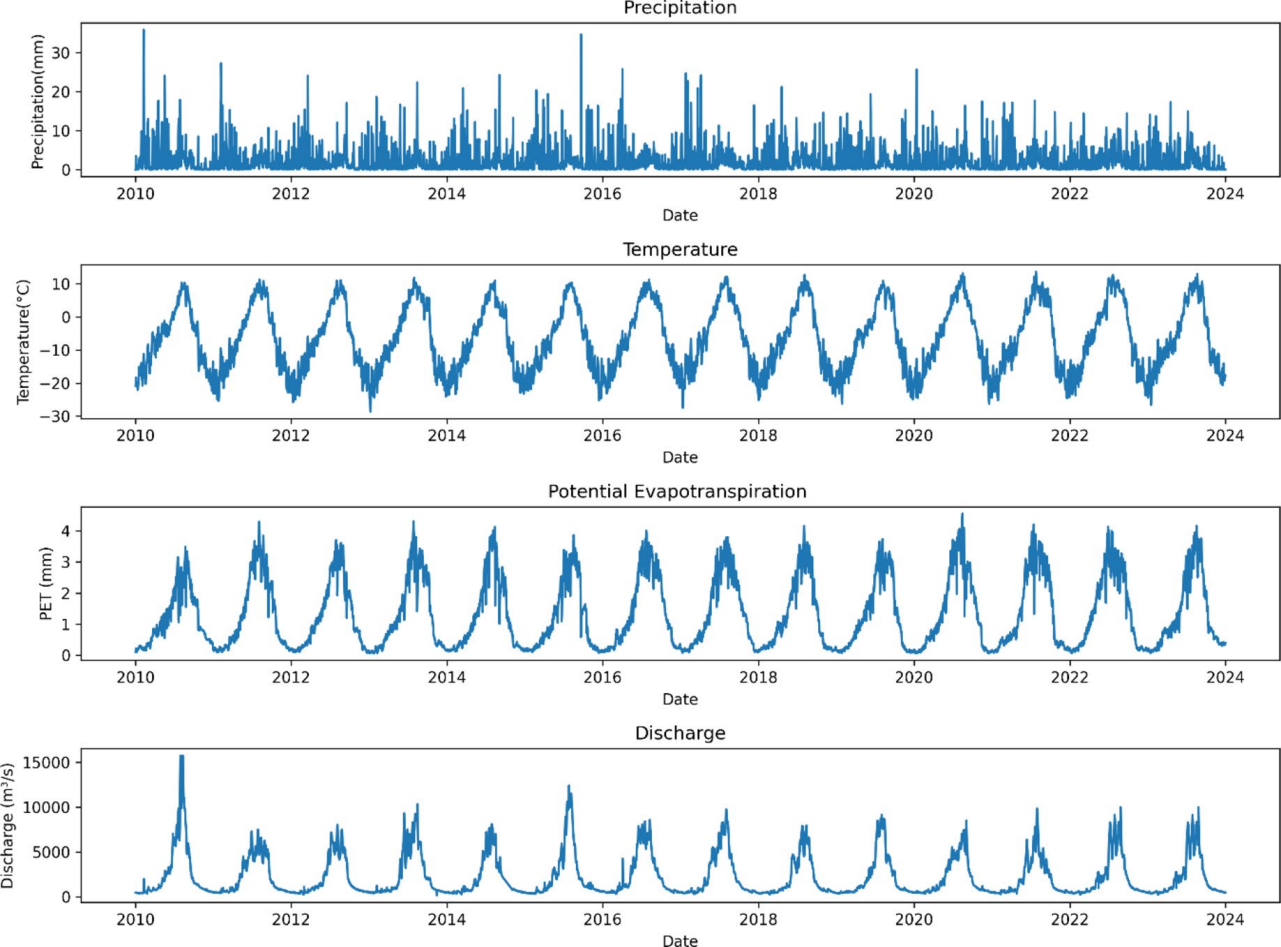
Daily data of precipitation, temperature and potential evapotranspiration and streamflow (discharge) in the Upper Indus Basin (UIB) used for calibrating (2010–2019) and validating (2020–2023) alternative model configurations.

This study employs three key datasets for hydrological simulation: daily precipitation, temperature, and potential evapotranspiration from ECMWF’s ERA5 (Fig. 2), and daily discharge data from the Water and Power Development Authority (WAPDA) in Pakistan, spanning from 01.01.2010 to 31.12.2023. These time series were divided into two subsets, the first for calibration (2010–2019) and the second for validation (2020–2023).

### Glacio-hydrological model

The GSM-SOCONT (Glacier-Snow Melt Soil CONTribution) model operates on water balance equations to predict daily streamflow^74^. This simulates various glacio-hydrological components including total runoff (Q), snowmelt discharge (Qsnow), snow water equivalent for the basin (SWEbasin), snow series (Sseries), precipitation equivalent for the basin (Peqbasin), baseflow (BF), evapotranspiration (ET), surface runoff (SR), total glacial melt discharge (Qgl_tot), glacial series (Gseries), snow water equivalent for glaciers (SWEgl), precipitation equivalent snowmelt for glaciers (PeqGl), and glacial melt (Qgl). The GSM-SOCONT model requires time series data for air temperature (T), precipitation (P) and potential evapotranspiration (PET) to simulate runoff. The output features from the GSM-SOCONT model are integrated into ML models to predict the discharge at the outlet of the UIB. By combining the strengths of the process-based glacio-hydrological model and data-driven ML approaches, this hybrid model can significantly improve the prediction of streamflow in complex environments like glacier-fed catchments, where traditional hydrological models may fail^75^.

**Fig. 3:**
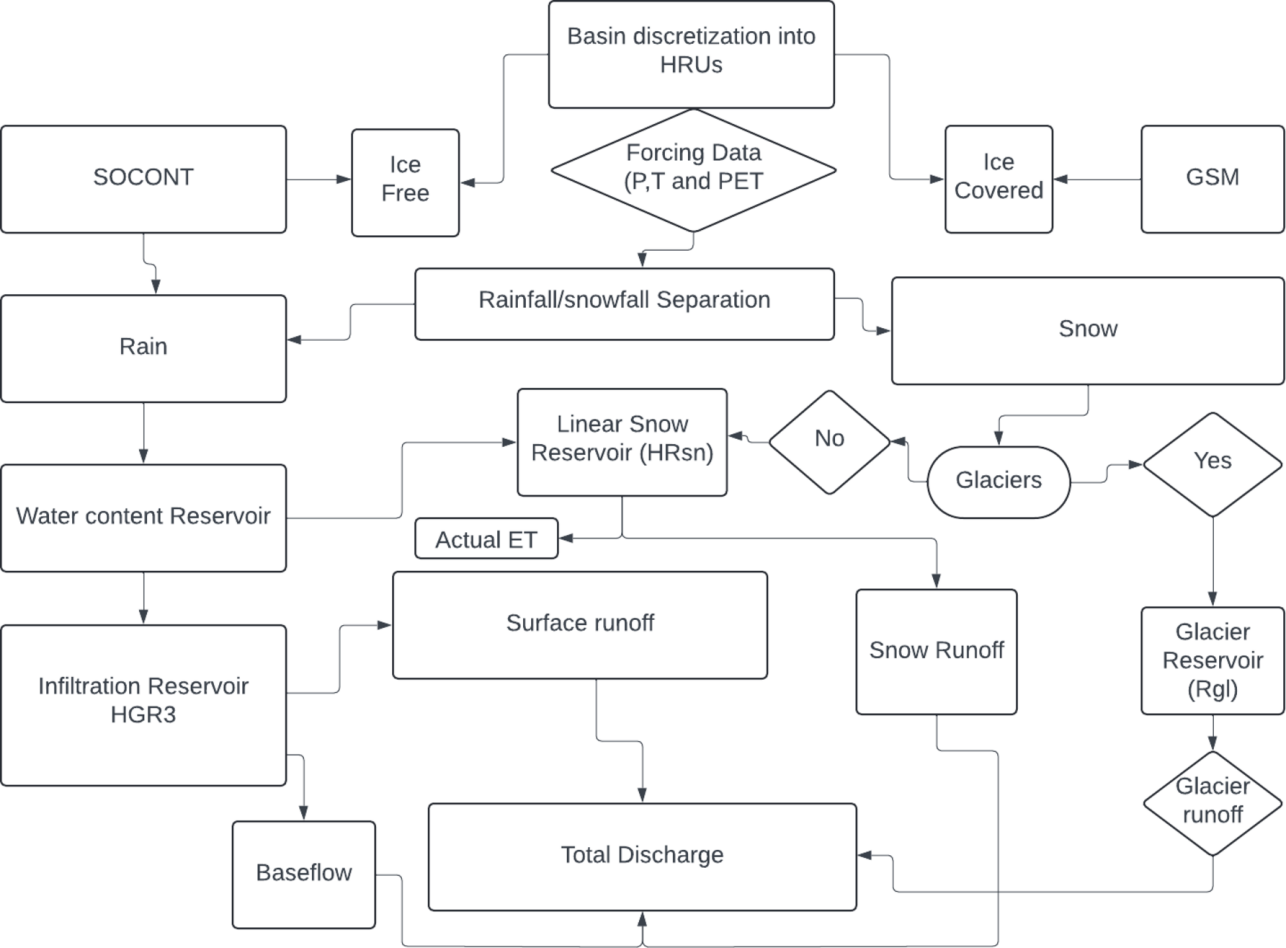
Flow diagram of the GSM-SOCONT model illustrating the basin discretization process and the application of the GSM (Glacier and Snow Melt) and SOCONT (Soil Contribution) components to simulate snowmelt, glacier melt, and rainfall-runoff processes across different sub-basins, integrating these outputs to predict overall streamflow in the catchment area.

A schematic of GSM-SOCONT^38^ is illustrated in Fig. 3. This semi-distributed conceptual model can account for various hydrological processes that affect river flows^76^. To account for the influence of temperature variations with altitude, basins are typically divided into elevation bands. When necessary, these elevation bands include a glacier melt model instead of a soil infiltration model.

In large catchments, the initial step often involves dividing the area into sub-basins before defining Hydrological Response Units (HRUs) or elevation zones. To account for the effects of elevation on temperature and hydrological processes, the UIB was divided into nine elevation zones consisting of nine sub-basins, resulting in 106 HRUs, of which 42 are area glacier-covered, with the remainder corresponding to non-glaciated areas.

In the absence of glaciers in the catchment area, SOCONT model calculates snow melt equivalent precipitation (Peq) and direct precipitation. Both snowmelt equivalent precipitation and direct precipitation are then processed through a non-linear soil infiltration reservoir based on GR3 equations^74,76^.

In the case of direct precipitation, surface runoff is simulated using the SWMM (Storm Water Management Model). The key parameter in SWMM is surface roughness, which affects generation and transportation of runoff across the surface. Contributions from each elevation band (discretized sections of the basin) are summed to determine the total discharge at the sub-basin outlet. This methodology provides a comprehensive approach to modelling river flow by integrating various hydrological processes and accounting for the effects of snow and glacier melt, soil infiltration, and surface runoff.

In parts of the UIB where glaciers were present, the SOCONT model was replaced by the glacier melt model (GSM) to accurately represent the contribution of glacier melt to runoff. The snow on glaciers is first melted and then glacial runoff is calculated based on temperature through a degree day equation. GSM consists of five sub-models to simulate the hydrological processes in snow-covered and glacierized catchments. The first two sub-models handle the snow processes while other three sub-models manage glacier processes. The snow model generates equivalent precipitation (Peq) and snow water equivalent (SWE). First, Peq is transferred to a linear snow reservoir (Rsn) which controls the storage and release of discharge from snow cover (Qsnow) using the release coeffiecient (Ksn) and glacier surface area using the Eqs. (1) and (2)

1 $$\:\frac{{dH}_{Rsn}}{dt}={P}_{eq}-{K}_{sn}\times\:{H}_{Rsn}$$

2 $$\:{Q}_{snow}={K}_{sn}\times\:{R}_{sn}\times\:A$$

Another sub-model simulates glacier melt once the snow cover on the glacier has fully melted. The resulting glacier melt (Qglacier) is routed to a linear glacier reservoir (Rgl), which regulates the storage and release of glacier runoff (Qglacier). This process is determined by the glacier melt reservoir level (HRgl) and the coefficient of the linear glacier reservoir (Kgl), as defined by Eqs. (3) and (4).

3 $$\:\frac{{dH}_{Rgl}}{dt}={P}_{eqGl}-\:{K}_{gl}\times\:{H}_{Rgl}$$

4 $$\:{Q}_{glacier}={K}_{gl}\times\:{H}_{Rgl}\times\:A$$

The Qsnow and Qglacier is combined at the outlet to produce total flow from ice cover area (Eq. 1).

5 $$\:Q={Q}_{snow}+{Q}_{glacier}$$

### Machine learning and deep learning

#### Random Forest Regressor (RFR)

Random Forests (RFs) is a robust, nonparametric method suitable for handling large, nonlinear, noisy, and multivariate data^77^. RFs is an ensemble method that combines multiple decision trees using bootstrap aggregating (or bagging) to improve predictive performance^78^. The bagging approach involves selecting random subsets from a training dataset to create multiple data samples, which are then used to train individual models independently. Instead of relying on a single decision tree, which might be unstable and prone to noise, RFs aggregate the results of many trees to make predictions more robust^79,80^. In addition, RFs also enhance the model predictive performance by randomly selecting subsets of features (variables) for each tree’s split points. Outputs from RFs are based on majority vote or averaging from all trees to make predictions. This ensemble approach is more robust compared to using a single decision tree.

#### eXtreme Gradient Boosting (XGBoost)

A key challenge in tree learning is finding the optimal split, which is addressed by eXtreme Gradient Boosting (XGBoost), an advanced version of gradient-boosted decision trees that delivers superior results^81^. Gradient Boosting is a powerful machine learning method used to enhance the performance of models by combining the strengths of multiple weak learners (often decision trees) to create a strong predictive model. In machine learning, two main types of errors are bias error and variance error^82^. The bias error occurs when a model is too simplistic and fails to capture the underlying patterns in data while variance error happens when a model is too complex and sensitive to fluctuations of the training data.

The XGBoost algorithm is particularly effective because it iteratively improves the model performance by focusing on the residual errors of previous models to capture more complex patterns and remining predictions^83^. XGBoost also incorporates Lasso and Ridge Regression regularization to penalize overly complex models and includes built-in cross-validation at each iteration to prevent overfitting. The distributed weighted quantile sketch algorithm within XGBoost helps to determine optimal split points and manage weighted datasets. Individual tree weights can be adjusted to reduce their influence on the final prediction. Its advantages include efficient tree pruning, parallel processing, and regularization. Additionally, features like shrinkage and column subsampling enhance the speed of computations in the parallel algorithm.

#### CNN-LSTM

In this study, Convolutional Neural Networks (CNN) and Long Short-Term Memory (LSTM) network were combined to predict streamflow. The CNN model developed by^84^ is primarily used for object recognition and consists of convolutional, pooling, and fully connected layers^85^. Convolutional layering applies filters to two-dimensional input data creating feature maps from input data, pooling layers reduce the dimensionality of these feature maps to speed up computation, and fully connected layers convert the feature maps into a one-dimensional vector. CNNs are used in digital image processing for object recognition^85^. To introduce non-linearity, an activation function like the rectified linear unit (ReLU) is used^86^. CNN is used to extract intrinsic features from the meteorological and discharge time series, while the LSTM uses these features for predicting discharge^58^. The integration of CNN and LSTM aims to leverage the ability of CNN model to handle nonlinear data for accurate streamflow predictions.

Deep learning models, particularly those with well-designed architectures, have shown strong performance in streamflow prediction^58,87^. Recurrent neural networks (RNNs), especially Long Short-Term Memory (LSTM) networks, are crucial in various fields such as speech recognition, image processing, and autonomous systems due to their effectiveness in modelling dynamic systems^88^.

The LSTM network (Hochreiter & Schmidhuber, (1997) is a type of recurrent neural network (RNN) designed to address issues like vanishing and exploding gradients while capturing long-term dependencies in sequences^89^. LSTMs, which use memory blocks in their hidden layers to manage input, output, and forget gates, are particularly adept at predicting non-linear, time-variant system outputs from time series data^90^. Each LSTM cell contains a memory unit and three gates - the input gate (It), the forget gate (Ft), and the output gate (Ot) - with linear equations involving weights (W) and biases (b) that vary at different steps^91^.

### Permutation feature importance and Wavelet transformation

The selection of an appropriate subset of environmental covariables is crucial for improving model efficiency^59^. Failing to select suitable covariables or using an excessive number can reduce the accuracy and stability of prediction models and increase memory and computational costs during training and validation^92^. Several techniques, such as permutation feature importance, SHAP (Shapley additive explanations), and graphical tool-based measures, have been introduced in ML to quantify feature importance based on contribution of each feature to the ML model prediction^93^. The permutation feature importance is used to estimate and assess feature importance and assist in interpreting individual features within complex frameworks, including deep neural networks, random forests, and support vector machines^94^.

The process of breaking down a function into wavelet components is called the wavelet transform^95^. Wavelet Transformation (WT) and Fourier series are mathematical tools increasingly used in hydrological and meteorological studies for time series analysis^96^. The Fourier series is a traditional method used to break down signals into their constituent frequencies. However, it has limitations, particularly in handling non-stationary data, where the statistical properties of the data change over time. Unlike the Fourier series, WT can simultaneously record both the time and frequency characteristics of a signal, making it more suitable for analysing complex, time-varying data. A wavelet is a mathematical function used to analyse a time series in both space and scale^97^.

Wavelet analysis comes in two forms like continuous wavelet transforms (CWT), and discrete wavelet transform (DWT). DWT is more commonly applied in hydrologic time-series forecasting because hydrological data are typically recorded at discrete time intervals^98^. In wavelet transform, the filtering algorithm split data into two sub-sets such as approximations and details. Approximations contain large-scale, low-frequency components, while details capture small-scale, high-frequency components. By breaking down the data into these subsets, the variance of the original data series is preserved, allowing the original data to be reconstructed by reversing the decomposition process^99^. During the first decomposition, the original signal is divided into approximations (A1) and details (D1) coefficients, functioning as high-pass and low-pass filters. The low-frequency component (A1) obtained can be further decomposed into A2 and D2, where the original signal is decomposed across multiple levels.

The wavelet decomposes time series using a function as in Eq. (6) as follows:

6 $$\:{\Psi\:}={2}^{-\frac{j}{2}}{\int\:}_{j}^{1}{\Psi\:}\left({2}^{-\frac{j}{2}-k}\right)f\left(t\right)\hspace{0.17em}dt$$

where ψ refers to the discrete wavelet transform in the time series shown by *f(t)*, *j* is the decomposition level, and *k* is the origin length.

### Hybrid model development scenarios

**Fig. 4:**
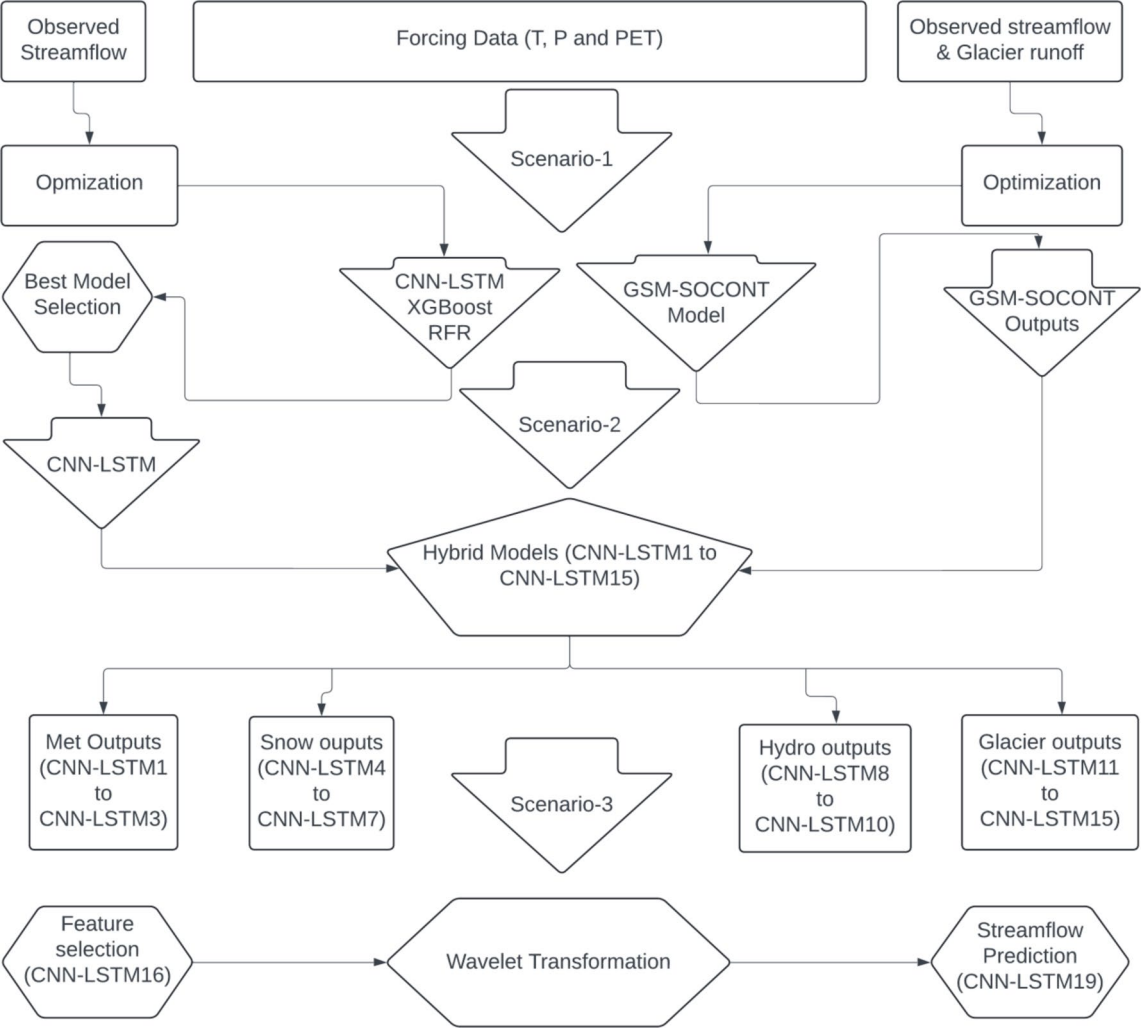
The flowchart depicts the methodology for evaluating streamflow predictions across three scenarios.

After calibrating the GSM-SOCONT model, its output features were then used as input features to a deep learning hybrid models (CNN-LSTM), leading to the creation of a series of hybrid models to predict the discharge at the outlet of the UIB (Fig. 4). By leveraging the strengths of glacio-hydrological model and data-driven models, a hybrid model can significantly improve the prediction of streamflow in complex environments like glacier-fed catchments where traditional physical models may fail^75^. This approach integrates physics-based predictors through the glacio-hydrological model, combining the interpretability and physical consistency of traditional models with the predictive power of ML. This methodology provides a robust solution for complex prediction tasks in hydrology and environmental studies. This methodology includes the following three scenarios:

Scenario 1. The first scenario benchmarks the performance of GSM-SOCONT and various machine learning (ML) models using only meteorological features such as temperature (T), precipitation (P), and potential evapotranspiration (PET). This baseline scenario helps to establish a reference point for evaluating how well the ML models perform relative to the GSM-SOCONT model and to choose the best ML/DL model.

Scenario 2. The second scenario incorporates the intermediate features generated by the GSM-SOCONT model into the best-performing ML/DL model from Scenario 1. These outputs include snowmelt discharge (Qsnow), snow water equivalent for the basin (SWEbasin), snow series (Sseries), precipitation equivalent for the basin (Peqbasin), baseflow (BF), evapotranspiration (ET), surface runoff (SR), total glacial melt discharge (Qgl_tot), glacial series (Gseries), snow water equivalent for glaciers (SWEgl), precipitation equivalent snowmelt for glaciers (PeqGl), and glacial melt (Qgl).

Scenario 3: The third scenario uses a permutation feature selection method to identify the most important input features for discharge prediction. These selected features are then processed with wavelet transformation to refine the runoff predictions from the catchment. This scenario is designed to test whether focusing on key predictive features and enhancing them through wavelet transformation can lead to better performance compared to the broader approaches used in scenarios 1 and 2.

The superior ability of CNNs to extract high-level spatial features from input data (e.g., temperature, precipitation etc.) combined with LSTMs strength in capturing temporal patterns from sequential data make them appropriate for predicting target variables (e.g., streamflow). Machine learning (ML) models (e.g., RFR and XGBoost) are powerful tools but they are not specifically designed to capture sequential dependencies, which are essential for accurate streamflow prediction. However, testing the performance of both machine learning models and neural networks could offer valuable insights into their respective abilities for accurate streamflow prediction. Furthermore, the use of wavelet transformation helps to improved prediction accuracy in streamflow prediction^42,100^. It allows the model to capture both short-term and long-term fluctuations by decomposing time-series data into different frequency components. Combining machine learning and wavelet transformation techniques may enhance the ability of the model to isolate and analyse features at multiple time scales, providing more accurate and robust prediction in snow and glacier-fed catchment of the UIB.

### Performance evaluation

In this study, the performance of the GSM-SOCONT and data-driven models was assessed using five metrics including Nash-Sutcliffe Efficiency (NSE; Nash & Sutcliffe^101^), Kling-Gupta Efficiency (KGE)^102^, Pearson Correlation Coefficient (R), Root Mean Square Error (RMSE) and Mean Absolute Error (MAE). In addition, the percentage bias for low flows (bottom 30%) (FLV) and high flows (top 2%) (FHV) was calculated to evaluate the performance of the model under non-monsoonal conditions and peak flow scenarios^103^. These metrics were employed to determine the relative performance of the stand-alone and coupled models. RMSE measures the average prediction error of the model by quantifying the difference between observed outcomes and the predicted values. A lower RMSE indicates a better model performance, as it reflects a smaller average discrepancy between the actual and predicted values. MAE is an alternative metric to RMSE that is less affected by outliers. A lower MAE indicates better model performance, as it reflects a smaller average deviation between the actual and predicted values.

The NSE quantifies the accuracy of the hydrological model, with values ranging from 1 to negative infinity; an NSE of 1 represents a perfect match between simulated and observed flows, while a negative NSE signifies poor model performance. The calculations for these criteria are detailed below.

7 $$\:\text{NSE}=1-\frac{{\sum\:}_{t=1}^{T}{\left({Q}_{t,o}-{Q}_{t,m}\right)}^{2}}{{\sum\:}_{t=1}^{T}{\left({Q}_{t,o}-\stackrel{-}{{Q}_{o}}\right)}^{2}}$$

Where $\:{Q}_{t,m}$ and $\:{Q}_{t,o}$ represent simulated and observed streamflow respectively, at time step t and $\:\stackrel{-}{{Q}_{o}}$ represents means of the observed streamflow.

The KGE equation is given by:

8 $$\:KGE=1-\sqrt{(r-1{)}^{2}+{(\alpha\:-1)}^{2}{+(\beta\:-1)}^{2}}$$

Where r is the correlation coefficient, α is the variability error and β is the bias error.

The RMSE and MAE are commonly used metrics for evaluating model performance. Given a sample of n observations y *(*$\:{y}_{i\:},i=\text{1,2},3,\ldots,n)$ corresponding model predictions $\:\widehat{y},\:$the MAE and RMSE are defined as follows:

9 $$\:RMSE=\sqrt{\frac{1}{n}}\sum\:_{i=1}^{n}({y}_{i}-{\widehat{y}}_{i}{)}^{2}$$

10 $$\:MAE=\frac{1}{n}\sum\:_{i}^{n}|{y}_{i}-{\widehat{y}}_{i}|$$

The equation for the Pearson correlation coefficient is:

11 $$\:R=\frac{{\sum\:}_{i}^{n}({x}_{i}-\widehat{x})({y}_{i}-\widehat{y})}{\sqrt{\sum\:_{i}^{n}({x}_{i}-\widehat{x}{)}^{2}.\:\sum\:_{i=1}^{n}({y}_{i}-\widehat{y}{)}^{2}}}$$

Where n is the number of observations, $\:{x}_{i}$ and $\:{y}_{i}$ are the sample points for variables x and y and $\:\widehat{x}$ and $\:\widehat{y}\:$are the means of x and y respectively.

## Results and discussion

### GSM-SOCONT model calibration and validation

In this study, the period from 01-01-2010 to 31-12-2019 was defined as calibration (training), while the period from 01-01-2020 to 31-12-2023 was designated as validation (testing) period. A simultaneous calibration approach was employed in GSM-SOCONT model, using a multi-objective strategy with equal emphasis on NSE, KGE, and R metrics. The calibration was conducted using the SCE-UA (Shuffled Complex Evolution-University of Arizona) method^104^.

The simulation of daily streamflow with GSM-SOCONT model at the outlet of the UIB was evaluated using several statistical metrics, as shown in Table 1. Both the calibration and validation periods displayed consistent and satisfactory performance, with the calibration period achieving NSE = 0.84, *R* = 0.93, and KGE = 0.85, while the validation period yielded NSE = 0.75, *R* = 0.92, and KGE = 0.75. These results suggest a reasonable level of accuracy for runoff simulation in a glacierized catchment. During calibration, the model demonstrates strong performance with high NSE, R, and KGE values, reflecting a good fit and precision in simulating observed runoff. However, high RMSE and MAE values reveal the presence of significant prediction errors. In the validation phase, the model maintains good performance with high NSE, R^2^, and KGE, though these metrics are slightly lower than in calibration period. The increased RMSE and MAE indicate that while the model remains effective, its performance diminishes somewhat when applied to unseen data.

The GSM-SOCONT model was calibrated based on daily discharge data at the outlet of the UIB, a standard approach for hydrological model calibration^17,105^. In regions where glacier mass balance or runoff data is available, the glacier-specific parameters can also be calibrated in glacio-hydrological models. In the GSM-SOCONT model, the calibration of degree-day factors was based on monthly glacier runoff estimates from the Python Glacier Evolution Model (PyGEM). This dataset includes projections of glacier mass balance, runoff, and related components, based on 22 general circulation models (GCMs) and various representative concentration pathways (RCPs), covering the period from 2000 to 2100. The use of glacier runoff data in this context is distinctive, as previous studies have not utilized it for glacier parameter estimation in the UIB. This approach helps in refining and eliminating unrealistic parameter values during the model calibration process, which has traditionally relied solely on discharge data.

**Table 1:** Performance metrics of glacio-hydrological model (GSM-SOCONT) during calibration (2010–2019) and validation (2020–2023) periods.

Metrics	Calibration	Validation
NSE	0.84	0.75
R	0.93	0.92
KGE	0.85	0.75
RMSE	989.5	1059
MAE	666.4	691.2

**Fig. 5:**
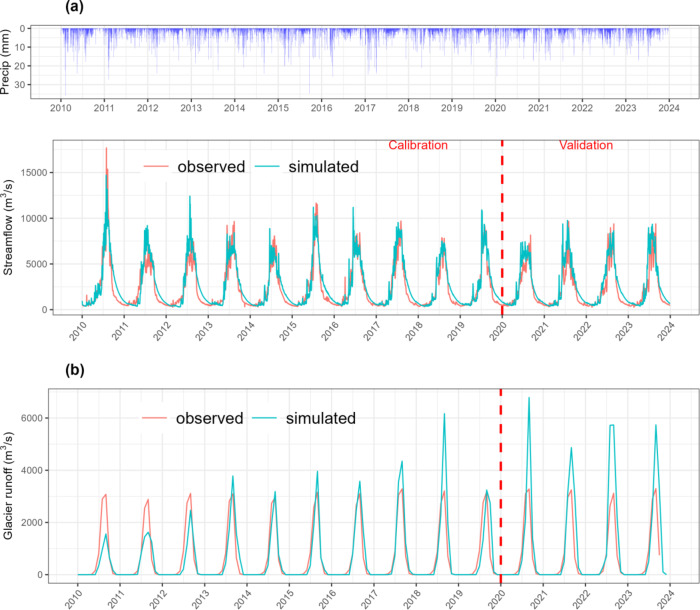
Model calibration and validation results for the Upper Indus Basin (UIB). (**a**) Comparison between observed and simulated daily streamflow during the calibration and validation periods, illustrating the performance of GSM-SOCONT in capturing peak flows and seasonal variations. (**b**) The monthly glacier runoff simulated with GSM-SOCONT after adjusting the glacier parameter values using PyGEM data, shows that the adjustments improved the accuracy of predicting glacier melt contributions during the calibration phase. However, during the validation phase, the model tended to overestimate glacier melt contributions.

Figure 5 presents the visual depiction of time series comparing daily measured runoff with simulated total runoff by the GSM-SOCONT during the study period (2010–2023). The hydrograph indicates that the GSM-SOCONT performs reasonably well, capturing peak flows but tended to overestimate low flows. Several factors, including geological, climatic, and topographic features and anthropogenic influences, can complicate low flows simulation^106–108^. Sapac et al.^109^ noted that hydrogeological conditions can influence the rainfall-runoff process and surface flow regime, making low-flow simulation particularly challenging in non-homogeneous catchments. Additionally, NSE is sensitive to peak flows which makes it less effective for simulating low flows and may lead to poor performance^110^. However, performance of the model is relatively accurate, still there could be areas for improvement. Predicting both high and low flows in a complex, glacierized basins remain a challenge, and the ability of the model to do so with reasonable accuracy is a positive outcome.

**Fig. 6:**
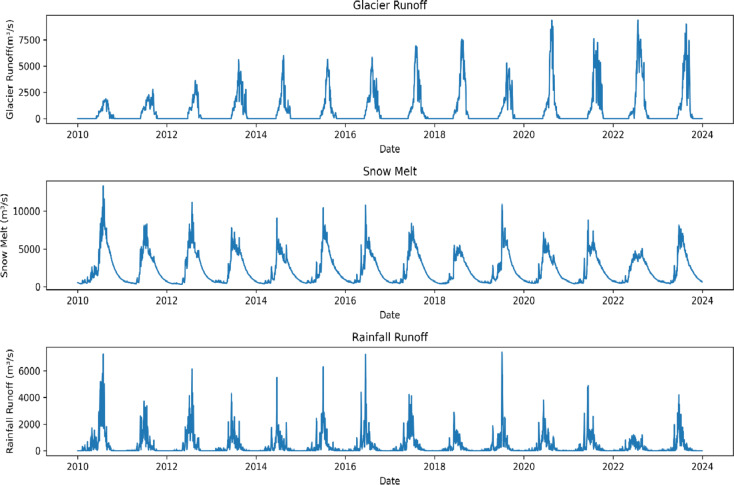
Proportional contributions of glacier, snowmelt, and rainfall to total runoff in the Upper Indus Basin (UIB).

Figure 5b illustrates the comparison of monthly observed and simulated glacier runoff. Initially, the GSM-SOCONT model was calibrated using only discharge data, which resulted in a reference degree day glacier melt coefficient (G) of around 0.5 mm/°C/day. By adjusting the glacier parameter values to approximately 10.6 mm/°C/day using data from the PyGEM model, the simulated glacier runoff better aligned with the PyGEM glacier runoff data (Fig. 5b). Figure 6 presents the relative contribution of daily glacier melt, snowmelt and rainfall after calibrating the model with discharge and glacier runoff data at the outlet of the UIB. The highest contribution of runoff comes from snowmelt (63.3%) than glacier melt (24.6%) and the least contribution is from liquid precipitation (11.96%) in the UIB.

### Machine learning model calibration and validation

**Fig. 7:**
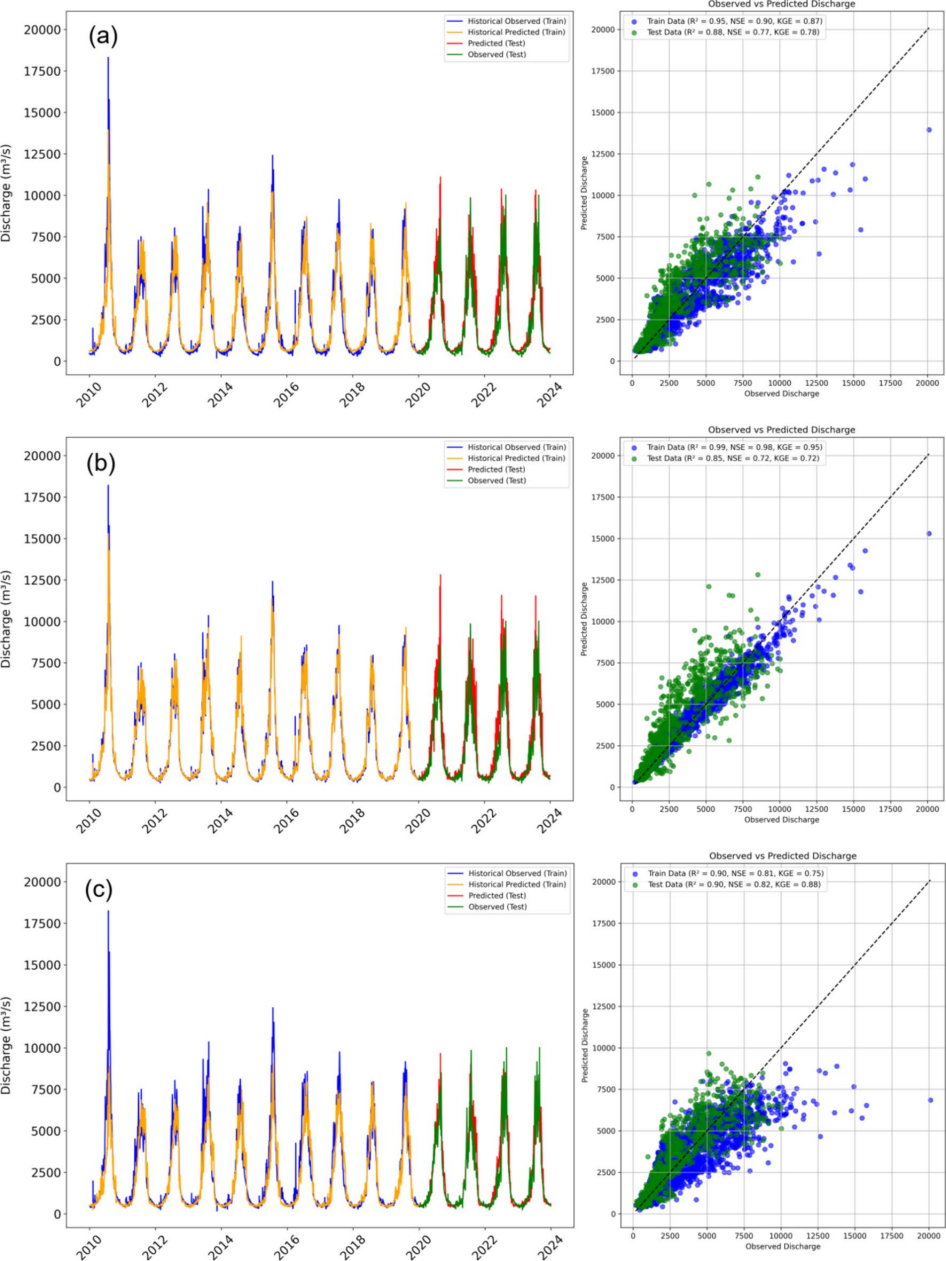
Line graphs and scatter plots illustrate the comparison between observed and simulated runoff using meteorological inputs for three machine learning models: (**a**) RFR, (**b**) XGBoost, and (**c**) CNN-LSTM. Each plot displays model performance across both the training period (2010–2019) and the testing period (2020–2023), highlighting the agreement between predicted and observed runoff values.

First, initial experiments were conducted to choose the best performing ML model through the calibration and validation process. For calibration and validation of machine learning (ML) models, the data was split into training (01-01-2010 to 31-12-2019) and testing (01-01-2020 to 31-12-2023) periods. The output features (see above Sect. 2.6) from glacio-hydrological were used to construct ML models for predicting target variable (e.g., streamflow) and to see how these features affect the accuracy of predictive models. These ML models were trained on calibration dataset using optimization algorithms to minimize error (e.g., RMSE, MAE). Tuning hyperparameters can significantly influence the performance of the model. Manually adjustments of these hyperparameters is impractical due to their large number. In this study, hyperparameter tuning was performed using grid search for RFR (n_estimators = 300, max_depth = None, min_samples_split = 2, min_samples_leaf = 1, bootstrap = true) XGBoost (n_estimators = 300, max_depth = 6, learning_rate = 0.1) and CNN-LSTM (learning rate = 0.01, optimizer = Adam, epochs = 620, batch size = 32, dropout rate = 0.2) models. The stochastic outputs in the LSTM model were managed by taking the average of predictions across multiple runs. To avoid overfitting, regularization and early stopping techniques were applied to ML models. The trained ML models were evaluated on validation data using performance metrics (e.g., NSE, KGE and R). The performance evaluation of RFR, XGBoost and CNN-LSTM hybrid models to simulated discharge is presented in Table 2. Figure 7 represents a graphical depiction of the performance metrics.

The CNN-LSTM hybrid model shows high performance with a relatively high NSE (0.81) and KGE (0.75) on the training data. It underperforms compared to GSM-SOCONT model which achieved higher NSE (0.84) and KGE (0.85) during calibration phase. However, CNN-LSTM hybrid model shows the higher performance during validation (NSE = 0.82 and KGE = 0.88) than GSM-SOCONT (NSE = 0.75, KGE = 0.75).

XGBoost exhibits the strongest training performance with NSE (0.98) and KGE (0.95), indicating very good fit along with high Pearson correlation coefficient (*R* = 0.99) values. However, there is a drop in performance during validation phase, with NSE (0.72) and KGE (0.72). This suggests overfitting, where the model performs well on training data but struggles with generalization to unseen data. XGBoost has the lowest RMSE and MAE on the training data, indicating precise predictions during training. RMSE and MAE of XGBoost are the highest on test data among the models, highlighting potential issues with overfitting.

**Table 2:** Performance metrics of machine learning and deep learning models during training and testing data.

Metrics	CNN-LSTM	RFR	XGBoost
Train NSE	0.81	0.9	0.98
Train KGE	0.75	0.87	0.95
Train R	0.9	0.95	0.99
Train RMSE	1092	379	353
Train MAE	598	212	221
Test NSE	0.82	0.77	0.72
Test KGE	0.88	0.78	0.72
Test R	0.9	0.88	0.85
Test RMSE	922	1147	1233
Test MAE	532	695	735

CNN-LSTM shows higher performance against RFR and XGBoost during validation. The Pearson correlation coefficient (R) values of CNN-LSTM hybrid model for both training and testing data (0.90) demonstrate a strong linear relationship between the observed and predicted streamflow. The RMSE and MAE are lower during testing period than the training, suggesting good generalization of CNN-LSTM. Scatter plot of CNN-LSTM show a deviation to the right side of the 1:1 line at higher runoff values which indicate that the model underestimate peak flows. Based on above metrics, the hybrid architecture of CNN-LSTM model proved to be the best choice for predicting streamflow using glacio-hydrological model outputs.

### Hybrid models to investigate effective inputs to predict streamflow

Table 3 outlines a systematic approach for evaluation of CNN-LSTM hybrid models with varying combinations of inputs derived from GSM-SOCONT model. The aim is to predict total discharge (Q) with different levels of complexity based on the specific group of input features like meteorological input features (PET, T, P), snow input features (Qsnow, SWEbasin, Sseries, Peqbasin), hydrological input features (BF, ET, SR), and glacier input features (Qgl_tot, Gseries, SWEgl, PeqGl, Qgl).

**Table 3:** Combinations of input and output variables from the GSM-SOCONT model for simulating runoff using CNN-LSTM model.

Sr.No	Input (s)	Outputs	Models
1	PET	Q	CNN-LSTM1
2	PET, T	Q	CNN-LSTM2
3	PET, T,P	Q	CNN-LSTM3
4	Qsnow	Q	CNN-LSTM4
5	Qsnow, SWEbasin	Q	CNN-LSTM5
6	Qsnow, SWEbasin, Sseries	Q	CNN-LSTM6
7	Qsnow, SWEbasin, Sseries, Peqbasin	Q	CNN-LSTM7
8	Baseflow	Q	CNN-LSTM8
9	Baseflow, ET	Q	CNN-LSTM9
10	Baseflow, ET, Surface runoff	Q	CNN-LSTM10
11	Qgl_tot	Q	CNN-LSTM11
12	Qgl_tot, Gseries	Q	CNN-LSTM12
13	Qgl_tot, Gseries, SWEgl	Q	CNN-LSTM13
14	Qgl_tot, Gseries, SWEgl, PeqGl	Q	CNN-LSTM14
15	Qgl_tot, Gseries, SWEgl, PeqGl, Qgl	Q	CNN-LSTM15

CNN-LSTM1 was run using only PET while CNN-LSTM3 used all meteorological features (PET, T and P). CNN-LSTM1 hybrid model has the lowest NSE (0.67), while CNN-LSTM3 has the highest NSE (0.80) (Supplementary Table 1). The CNN-LSTM3 hybrid model proved to be more accurate during calibration with the highest NSE which suggests that it predicts better high flows on unseen data compared to CNN-LSTM1 (0.70) and CNN-LSTM2 (0.77) hybrid models. CNN-LSTM1 to CNN-LSTM3 hybrid models show a significant improvement in NSE (0.70 to 0.82) during validation. This indicates a progression in model performance, with CNN-LSTM3 emerging as the best-performing hybrid model across all metrics during calibration and validation phases.

**Fig. 8:**
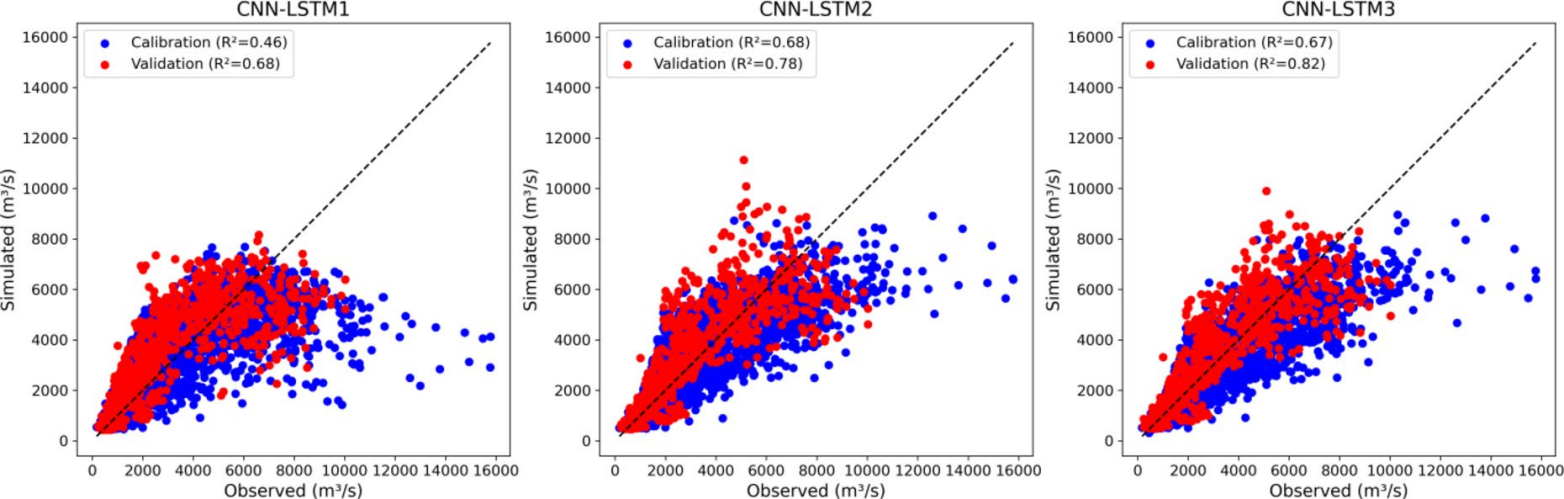
Scatter plots comparing simulated versus observed runoff from CNN-LSTM models based on glacier variables, with R^2^ values shown for both calibration and validation periods.

Figure 8 illustrates a significant increase in prediction accuracy during calibration and validation phases by an additional feature from CNN-LSTM1 to CNN-LSTM2. Further addition of feature in CNN-LSTM3 decreases the prediction accuracy (R^2^ = 0.68 to R^2^_=_0.67) slightly during calibration while a small increase during validation (R^2^ = 0.82). All scatter plots show deviation to the right side and more skewed towards the bottom of the 1:1 line which indicate that model underestimate peak flows during calibration and validation. The hybrid models demonstrate significant discrepancies in simulating both low flow volumes (FLV) and high flow volumes (FHV). CNN-LSTM1, CNN-LSTM2 and CNN-LSTM3 overestimates FLV by ~ 23%, 25.25% and 28.55%, while underestimates FHV by ~ 34%, 26.78% and 26% respectively.

**Fig. 9:**
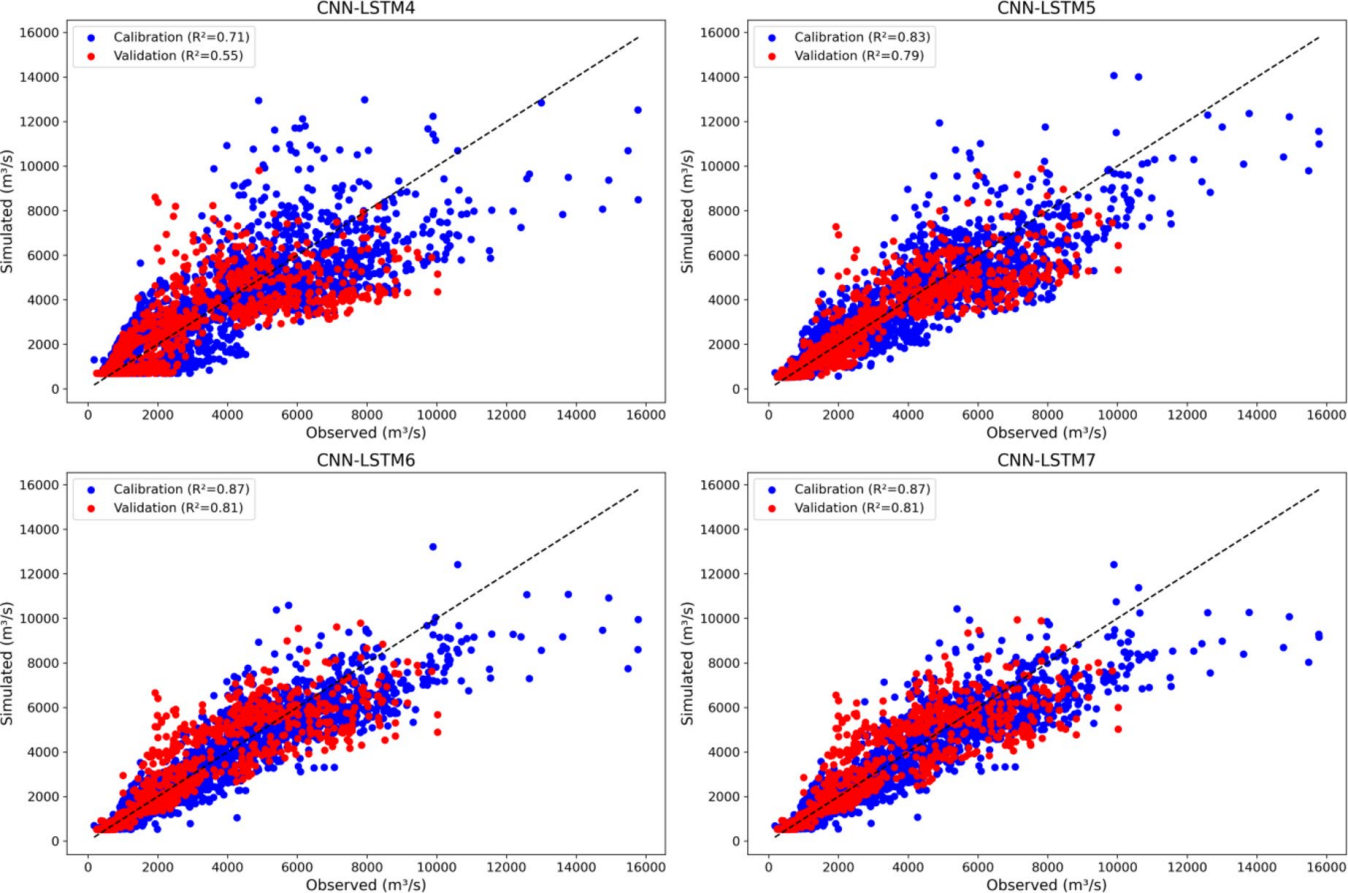
Scatter plots comparing simulated versus observed runoff from CNN-LSTM models based on glacier variables, with R^2^ values shown for both calibration and validation periods.

Figure 9 shows scatter plots of four CNN-LSTM models (CNN-LSTM4 to CNN-LSTM7) using snow outputs from the GSM-SOCONT model during the calibration and validation phases. CNN-LSTM7 exhibits the highest NSE at 0.90, indicating the best predictive accuracy, followed closely by CNN-LSTM6 (NSE = 0.89) hybrid model. CNN-LSTM4 has the lowest NSE (0.77) which makes it the least accurate among the four hybrid models in predicting high flows. Similarly, a significant decrease in error metrics (MAE and RMSE) from CNN-LSTM4 (MAE = 790, RMSE = 1195) to CNN-LSTM7 (MAE = 427, RMSE = 797) also confirm that CNN-LSTM7 hybrid model provides more accurate predictions during calibration.

The CNN-LSTM models show a consistent trend of overestimating low flow volumes (FLV) and underestimating high flow volumes (FHV). Specifically, CNN-LSTM4 exhibits the largest overestimation of FLV (69.68%) and underestimation of FHV (23.42%). Other models (CNN-LSTM5, CNN-LSTM6, and CNN-LSTM7) also overpredict low flows by around 30% and underpredict high flows by 17–18%, indicating a common challenge in accurately simulating both extremes in streamflow.

A systematic increase in performance indicators e.g., NSE (0.77 to 0.90) and KGE (0.83 to 0.87) from CNN-LSTM4 to CNN-LSTM7 indicates that incorporating additional features into the hybrid model has enhanced its ability to simulate streamflow accurately (Supplementary Table 2). The results show that systematic increase in additional features provide more information and context for hybrid models, allowing them to capture more complex patterns and relationships in the data. CNN-LSTM6 and CNN-LSTM7 both achieved the highest R (0.95) values which show that these models can explain 95% of the variance in the observed data. Whereas CNN-LSTM5 hybrid model leads in KGE (0.88), indicating a strong balance among correlation, variability bias, and mean bias.

CNN-LSTM5 has the highest NSE (0.84) which show its strong ability to predict high flows on unseen data. However, the lowest NSE (0.68) and KGE (0.76) of CNN-LSTM4 hybrid model suggests a significant drop in its predictive accuracy on unseen data. The highest KGE observed in CNN-LSTM6 and CNN-LSTM7 (0.90) suggests that these models maintain a good balance between correlation, variability, and bias. The performance of hybrid models is not increased with an additional feature from CNN-LSTM6 to CNN-LSTM7. This suggests that up to a certain point, additional features are beneficial simply but adding more features beyond a certain number does not necessarily lead to better performance. CNN-LSTM5, CNN-LSTM6, and CNN-LSTM7 all achieved a higher R value (0.91) during validation.

This analysis shows that CNN-LSTM7 is the best-performing model during calibration, with the highest NSE, KGE and R and lowest RMSE and MAE. CNN-LSTM5 emerges as the top hybrid model with the highest NSE, lowest RMSE and MAE, suggesting it generalizes better to new data compared to the other hybrid models. In conclusion, while CNN-LSTM7 shows the best performance during calibration, CNN-LSTM5 proves to be the most effective model in predicting unseen data, making it the preferred choice for applications requiring high generalization capability.

**Fig. 10:**
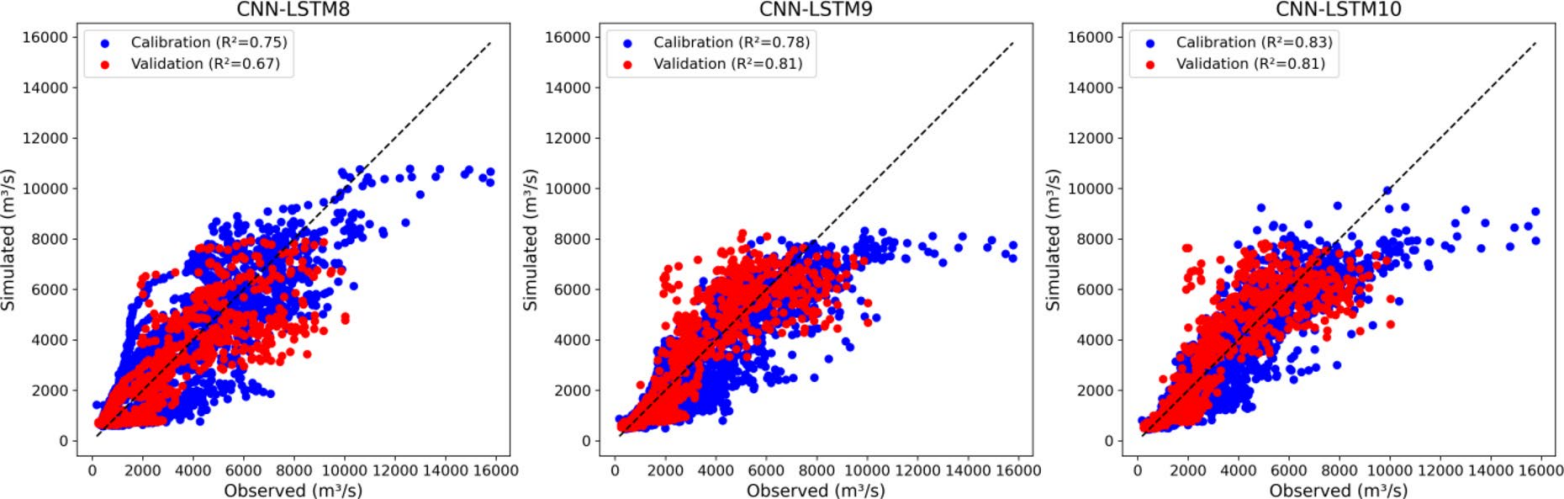
Scatter plots comparing simulated versus observed runoff from CNN-LSTM models based on glacier variables, with R^2^ values shown for both calibration and validation periods.

Figure 10 shows scatter plots for three CNN-LSTM hybrid models (CNN-LSTM8, CNN-LSTM9, and CNN-LSTM10) during the calibration and validation phases based on hydrological outputs (Baseflow, Evapotranspiration and Surface runoff) from GSM-SOCONT model. CNN-LSTM10 hybrid model achieves the highest NSE (0.86) and R (0.93) and the lowest RMSE (916) and MAE (515) during calibration phase (Supplementary Table 3). CNN-LSTM8 hybrid model may have a better balance among correlation, variability bias, and mean bias due to its highest KGE (0.86) despite it is less accurate overall.

CNN-LSTM9 and CNN-LSTM10 both achieved the highest NSE (0.81), KGE (0.90), R (0.90) and the lowest MAE (537) on unseen data. CNN-LSTM10 emerges as the best-performing model during the validation phase, indicating strong predictive accuracy and reliability. However, CNN-LSTM9 slightly edges out CNN-LSTM10 with marginally better RMSE. Overall, CNN-LSTM9 demonstrates the best balance between calibration and validation performance, making it the most robust model among the three hybrid models for predicting discharge at the outlet of the UIB.

The results of the CNN-LSTM8 to CMM-LSTM10 models indicate varying performance across low and high flow scenarios. CNN-LSTM8 significantly overestimated FLV by 78.45% and moderately underestimated FHV by 17.69%. In contrast, CNN-LSTM9 demonstrated improved FLV predictions with a 33.71% overestimation, but its FHV predictions had a larger underestimation of 20.08%. CNN-LSTM10 further improved FLV accuracy with a 29.33% overestimation and showed the smallest FHV underestimation at 18.31%, suggesting it provides the most balanced performance among the three models.

**Fig. 11:**
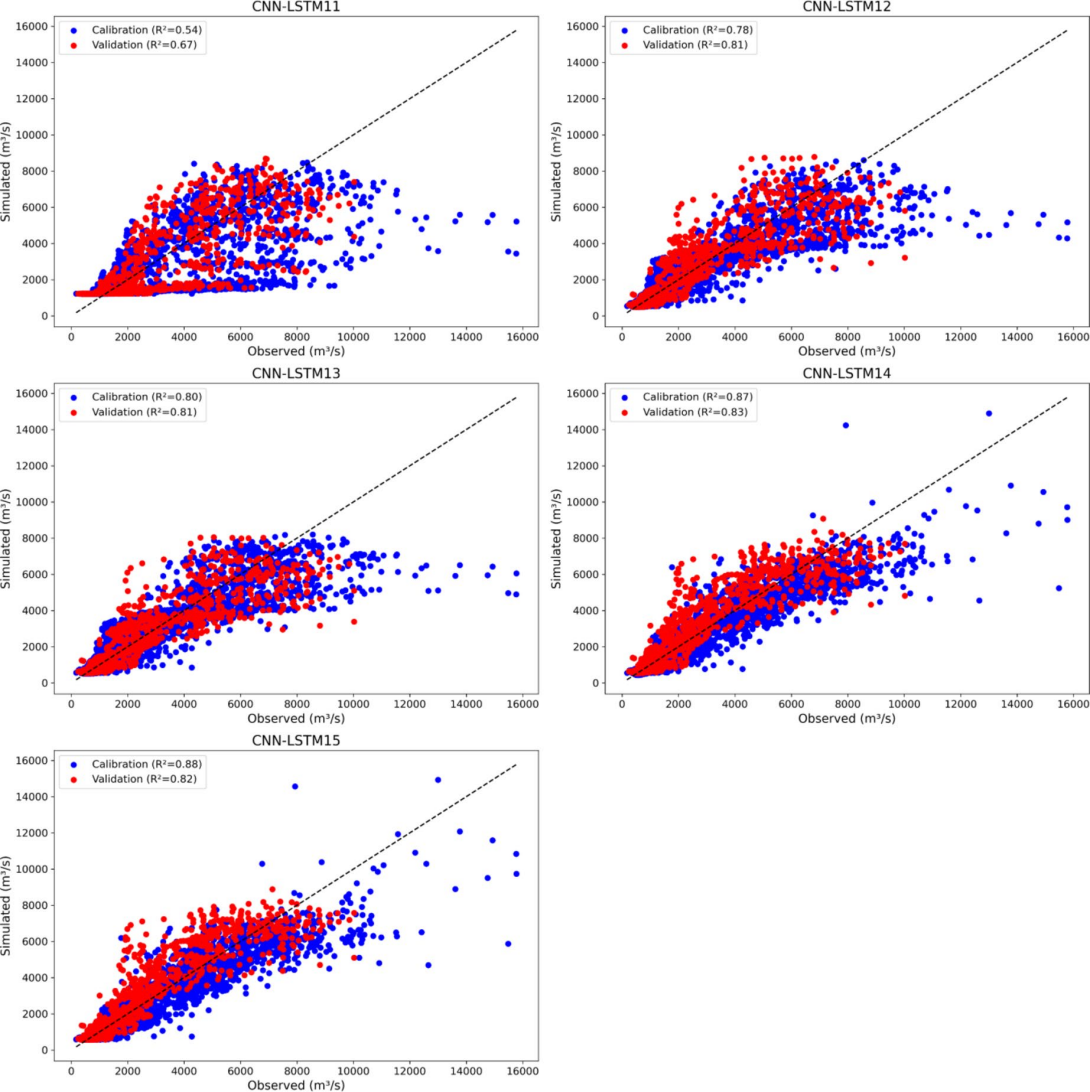
Scatter plots comparing simulated versus observed runoff from CNN-LSTM models based on glacier variables, with R^2^ values shown for both calibration and validation periods.

Figure 11 illustrates scatter plots of CNN-LSTM11 to CNN-LSTM15 during the calibration and validation phases. CNN-LSTM15 shows the highest NSE (0.87), indicating the best predictive accuracy during calibration while, CNN-LSTM11 has the lowest NSE (0.53) (Supplementary Table 4). The NSE values progressively improve from CNN-LSTM11 to CNN-LSTM15, indicating a trend of increasing model performance with additional features. The KGE also follows a similar trend, with CNN-LSTM15 achieving the highest KGE (0.83). Both CNN-LSTM14 and CNN-LSTM15 have the highest correlation (*R* = 0.93), while CNN-LSTM15 has the lowest error metrics (RMSE = 901, MAE = 500) confirming its higher accuracy in predictions.

During validation, CNN-LSTM14 achieves the highest NSE (0.83) compared to CNN-LSTM12 to CNN-LSTM15, indicating they are also reliable but slightly less accurate than CNN-LSTM14. The highest correlation during validation is observed with CNN-LSTM14 (*R* = 0.91) and has the lowest RMSE during validation (892). CNN-LSTM15 performs exceptionally well during calibration, with the highest NSE, KGE, R, and lowest RMSE and MAE, whereas CNN-LSTM14 emerges as the most robust model, particularly in terms of validation performance, making it the most reliable for predictions on unseen data.

The CNN-LSTM11 to CNN-LSTM15 models show a wide range of performance for low and high flow predictions. CNN-LSTM11 significantly overestimated FLV by 190.75% and underperformed in FHV prediction with a 34.99% underestimation. Models CNN-LSTM12, CNN-LSTM13, CNN-LSTM14, and CNN-LSTM15 displayed more balanced results, with overestimations of FLV ranging from 34.79 to 45.90% and FHV underestimations between 17.85% and 26.98%. These results suggest that while these models can simulate high flow volumes relatively better, their predictions for low flows are still subject to considerable overestimation, particularly for CNN-LSTM11.

**Fig. 12:**
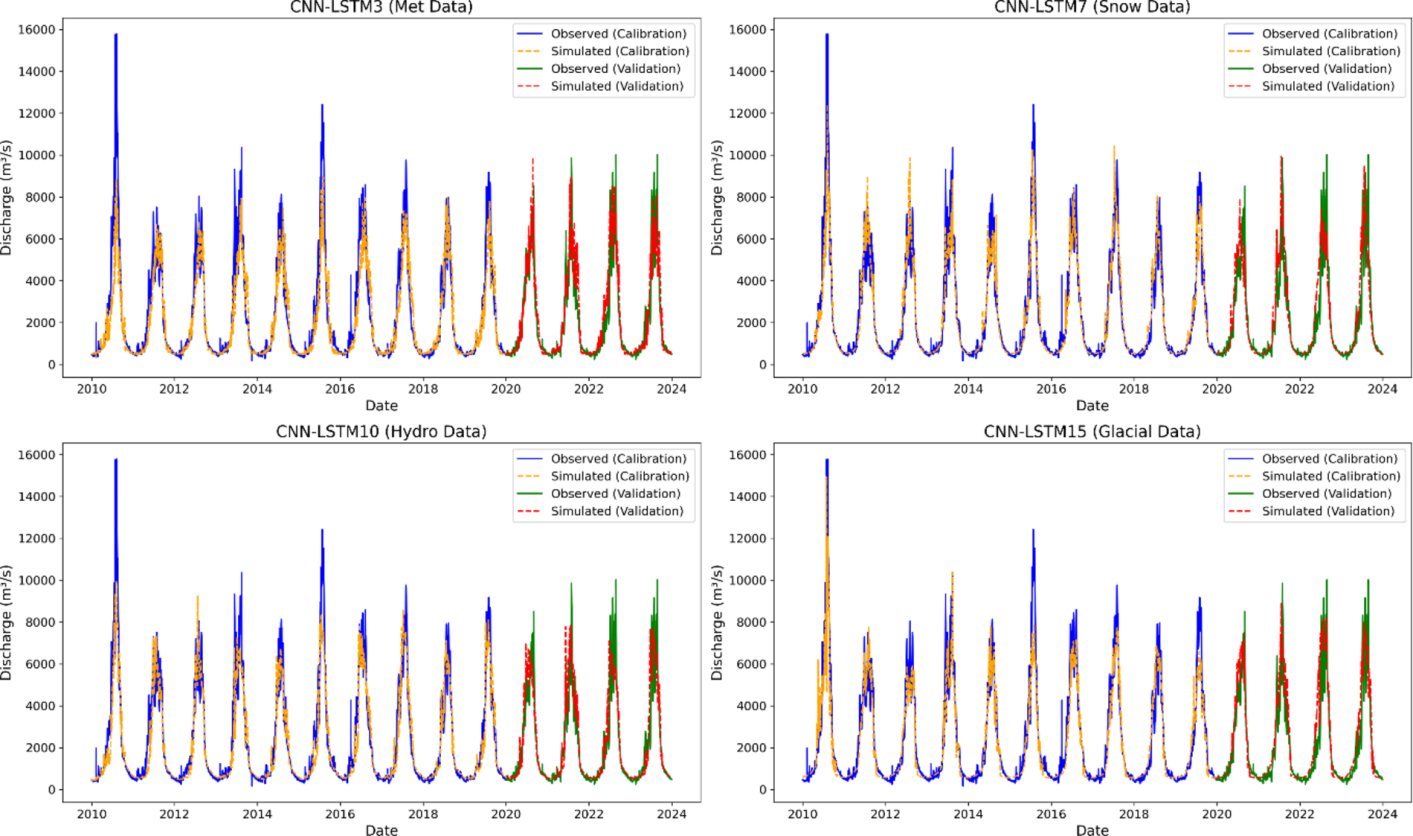
Line plots comparing simulated and observed runoff using the best hybrid models, incorporating meteorological, snow, hydrological, and glacial input features for simulating streamflow during calibration and validation periods.

Figure 12 presents the comparison of the performance of hybrid models using groups of output features from the GSM-SOCONT model. The best performing hybrid models from each group are CNN-LSTM3 using meteorological features, CNN-LSTM7 using snow output features, CNN-LSTM10 using hydrological output features, and CNN-LSTM15 using glacier output features (Fig. 12). Although CNN-LSTM15 excels during the calibration phase with the highest NSE, KGE, and lowest RMSE and MAE, CNN-LSTM14 proves to be more reliable for unseen data, making it the most robust model for predictions. It demonstrates superior generalization capability, especially during the validation phase, with the highest NSE (0.83), highest correlation (R of 0.91), and the lowest RMSE (892). CNN-LSTM9 also shows a strong balance between calibration and validation performance, particularly in hydrological predictions, making it second topmost contender.

### Feature selection and wavelet transformation

**Fig. 13:**
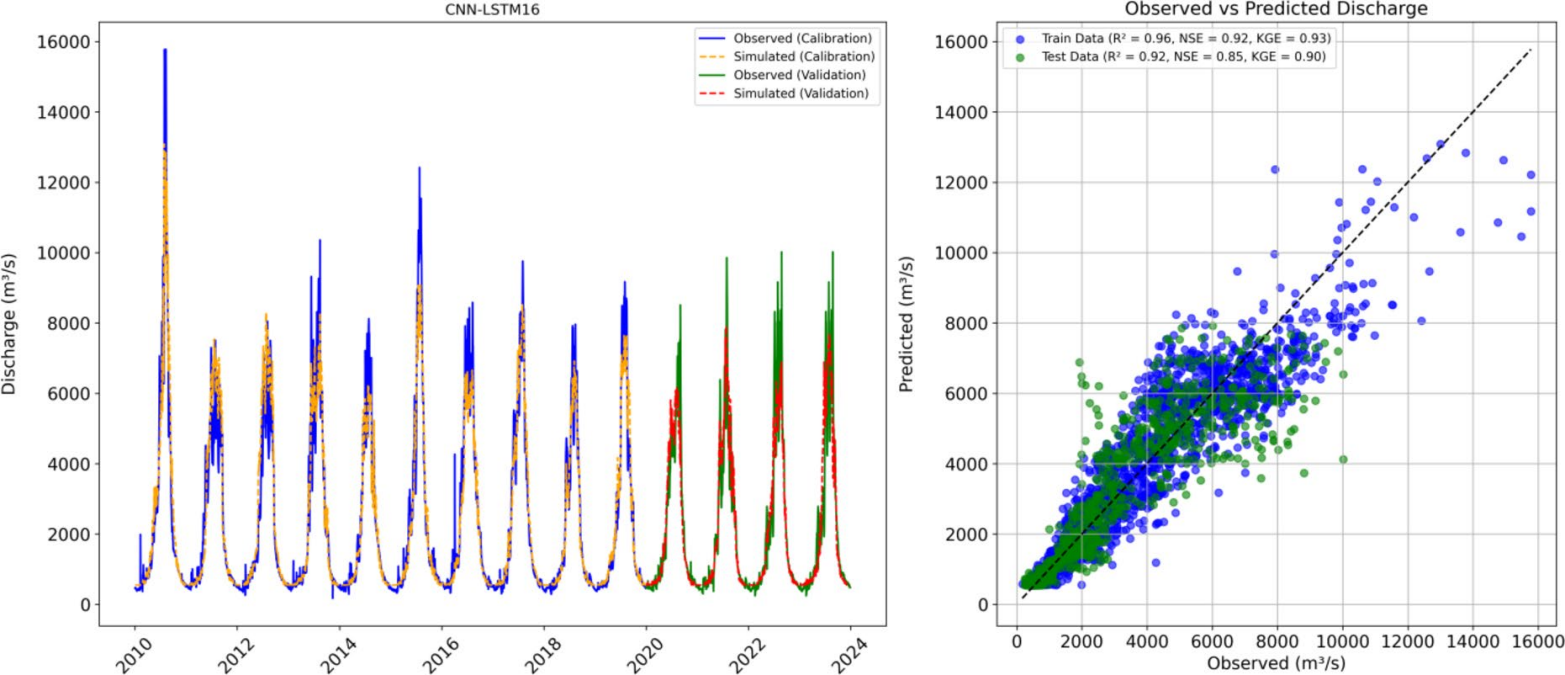
Line plot comparing simulated and observed runoff with the best hybrid model (CNN-LSTM16), which incorporate selected meteorological, snow, hydrological, and glacial features for discharge prediction during both calibration and validation periods.

Figure 13 illustrates the observed and predicted discharge during calibration and validation period using a hybrid model based on permutation feature importance method. The permutation feature importance method is used to select key features after evaluating different combinations of variables to construct fifteen hybrid models (CNN-LSTM1 to CNN-LSTM15). The key features including potential evapotranspiration (PET), temperature (T), snowmelt discharge (Qsnow), snow water equivalent for the basin (SWEbasin), baseflow (BF), surface runoff (SR), total glacial melt discharge (Qgl_tot), snow water equivalent for glaciers (SWEgl) and precipitation equivalent snowmelt for glaciers (PeqGl) were chosen using permutation feature importance method to develop another hybrid model (CNN-LSTM16). The results show a significant improvement in prediction accuracy of the newly developed hybrid model CNN-LSTM16. Previously, CNN-LSTM14, which utilized glacier output features was identified as the best hybrid model with strong validation phase metrics. CNN-LSTM16 hybrid model based on the selected features further enhanced the performance, increasing the NSE from 0.83 to 0.85, KGE from 0.88 to 0.90 and R from 0.91 to 0.92.

Additionally, three hybrid models were developed using selected features to evaluate the effectiveness of wavelet transformation in improving streamflow predictions. The features were decomposed into two levels using Symlet, Daubechies (db), and Coiflet (coif) filters, as suggested in previous studies^95^. Discrete wavelets transform (DWT) was applied to decompose the time series data into sub-series representing different frequency bands, including approximation (low-frequency) and detail (high-frequency) components. This decomposition helps the model focus on specific patterns within each frequency range, reducing noise and improving prediction precision. CNN-LSTM17, CNN-LSTM18, and CNN-LSTM19 hybrid models were then trained on the transformed and scaled data with optimized parameters to minimize prediction error. The convolutional layers captured localized patterns such as abrupt changes in streamflow or time trends.

CNN-LSTM19 achieved the highest NSE (1.00), indicating a near-perfect match between predicted and observed values during calibration period (Supplementary Table 5). CNN-LSTM19 outperforms the others with a KGE of 0.99, and lowest RMSE (143) and MAE (69). Similarly, CNN-LSTM19 has the highest NSE (0.96) indicating strong performance during validation phase. In addition, CNN-LSTM19 also achieved the best KGE (0.96), the highest R (0.98) and the lowest RMSE (442) and MAE (229) during validation phase. This suggests that CNN-LSTM19 is not only highly accurate during calibration but also generalizes well to unseen data.

The CNN-LSTM17 to CNN-LSTM19 models showed improvements in predicting FLV and FHV compared to earlier hybrid models. CNN-LSTM19 performed the best, with overestimation of FLV only 3.53% and underestimation of FHV only 2.14%. CNN-LSTM18 also achieved good results, overestimating FLV by 7.95% and underestimating FHV by 4.90%. CNN-LSTM17 displayed slightly higher errors, with a 9.27% overestimation for FLV and a 7.18% underestimation for FHV. These hybrid models indicate improved accuracy, particularly for CNN-LSTM19, which provides the most balanced predictions.

**Fig. 14:**
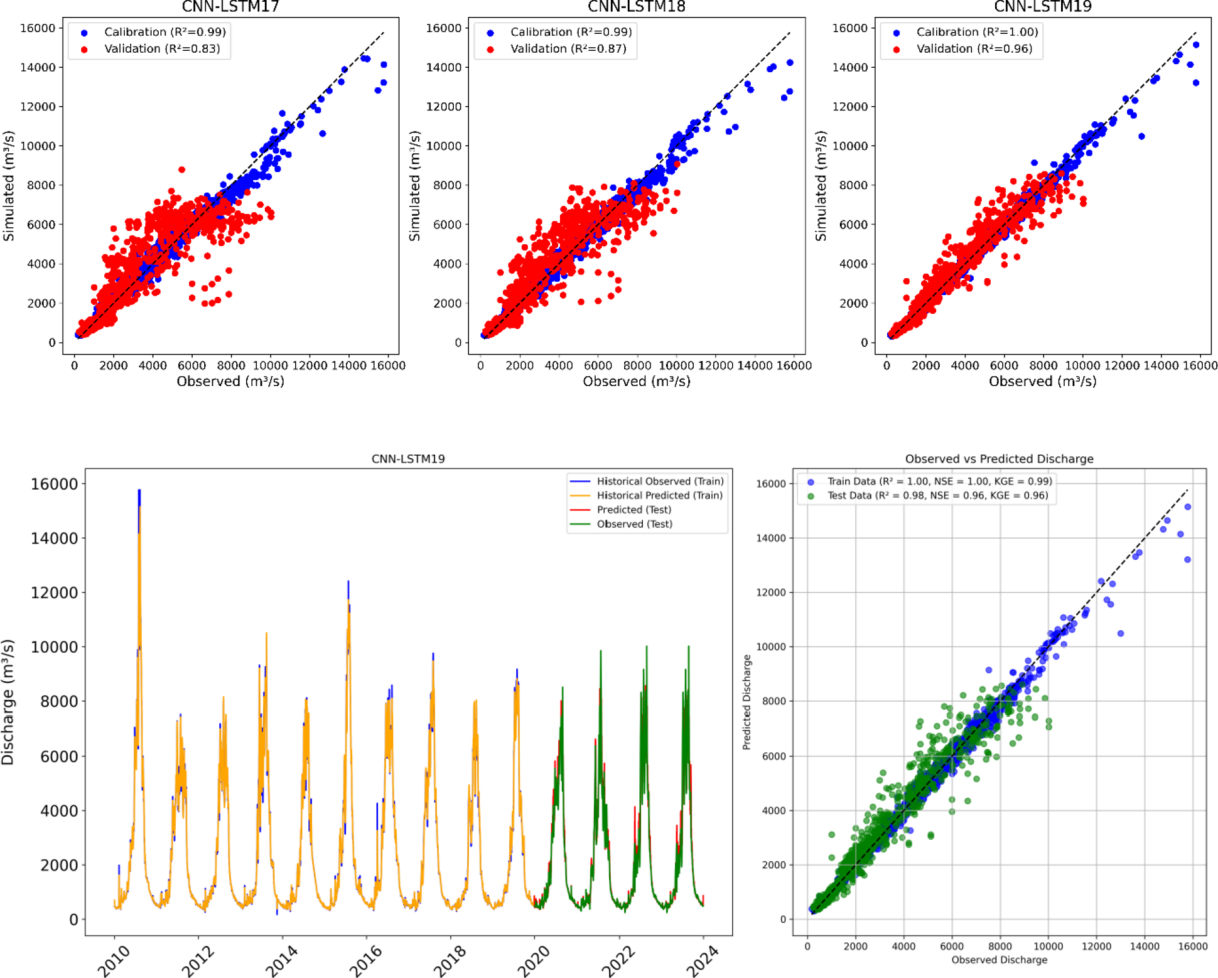
Scatter plots comparing simulated and observed runoff from CNN-LSTM models based on wavelet-transformed decomposed data, with the lower panel displaying a line plot of the best-performing hybrid model (CNN-LSTM19).

Figure 14 presents the scatter plots of hybrid models after applying wavelet transformations. The scatter plot of CNN-LSTM19 shows that most data points align closely with the 1:1 line, indicating that the model has achieved the highest accuracy with minimal bias. This alignment suggests that the model effectively captures both low and high flows and accurately predicting streamflow across the range of conditions. The corresponding line graph further supports this, showing that the model reliably captures peak flows and lower flows during both the calibration and validation periods, highlighting a strong performance in reproducing the observed streamflow patterns.

**Fig. 15:**
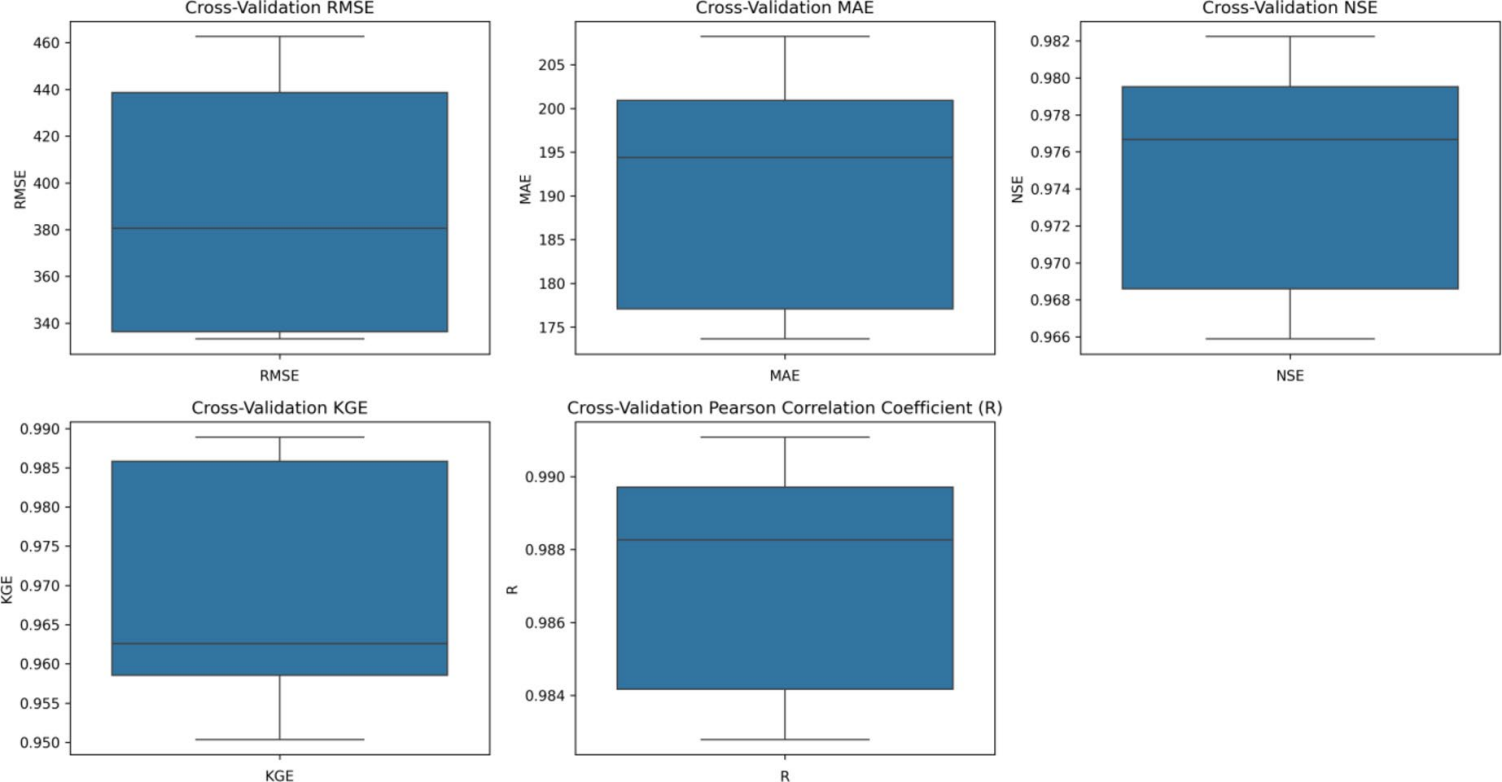
Boxplots of performance metrics for the CNN-LSTM19 model showing the results of 5-fold cross-validation for both training and test datasets.

The performance of the top-performing hybrid model (CNN-LSTM19) is validated using the k-fold cross-validation method. Figure 15 illustrates the results of a 5-fold cross-validation conducted on both the training and test datasets. Cross-validation is a technique used to evaluate the performance of a machine learning model by dividing the dataset into multiple subsets (folds), where the model is trained on some folds and tested on the remaining ones. The dataset is split into five equal parts (folds) (Supplementary Table 6). In each iteration, 4-folds are used for training, and the remaining 1-fold is used for validation (testing). This process is repeated five times, each time using a different fold as the validation set. The average of the performance metrics across all folds gives a robust estimate of ability of the model to perform on unseen data.

Across the five-folds (k1 to k5), the model consistently shows higher NSE values (0.97 to 0.98) and KGE values (0.95 to 0.99). The RMSE ranges between 333 and 462, with MAE values between 174 and 208. The model performs exceptionally well on training data, with all metrics indicating high predictive accuracy and reliability. The test data metrics show a similar trend, but slightly reduced performance compared to the training data, which is typical due to the absence of overfitting. The RMSE ranges from 362.45 to 453.85, and MAE from 187.27 to 224.17, indicating that the model remains consistent even on unseen data. Fold k3 shows the best performance on test data with the lowest RMSE (362.45) and MAE (190.89), and the highest NSE (0.9717) and R (0.9857). High NSE, KGE, and R values across all folds in both training and test data highlights the robustness and ability of CNN-LSTM19 hybrid model to capture the underlying patterns in the data.

## Discussion

The UIB hosts some of the largest glacier systems in the world outside the polar regions, including the Karakoram and western Himalayas^111^. Glaciers act as frozen water reservoirs, releasing water during the warmer months when snowmelt alone might not be sufficient. The hydrological regime in the UIB is governed by glaciers, snow, winter and monsoon rainfall^112^. Therefore, accurate simulation of these melt processes is crucial for understanding water availability, managing flood risks, and predicting how climate change may affect the region. The peak runoff occurs typically in a critical 5–8 week window during spring and summer months when increase in temperature accelerates the melting of snow and glaciers. This study emphasizes the significant improvement in streamflow forecasting achieved by integrating a glacio-hydrological model with machine learning. It also highlights the effectiveness of hybrid models, particularly those combining features from the GSM-SOCONT model with CNN-LSTM, surpassing standalone methods.

Glacier melt is the second largest contributor to the total runoff (Fig. 6). Our findings align with results from previous studies conducted across various UIB catchments. For instance, in the Hunza River Basin and Chotta Shigri Glacier, snowmelt constitutes half of the total annual runoff, followed by glacier melt (about one-third), with the remaining attributed to rainfall^25,113^. In contrast, the upper Ganges and Brahmaputra exhibit lower contributions, ranging from 10 to 20% of their annual streamflow. Biemans et al.^114^ highlighted a substantial contribution of snow and ice melt, constituting approximately 60–70% of the total runoff. More than 80% of sno and glacier melt contribution in the UIB originates from less than 20% of itsarea^115,116^. The relative contributions of snowmelt, glacier melt, and rainfall to the total runoff in the UIB were quantified, with snowmelt contributing 63.3%, glacier melt 24.6%, and rainfall 11.96%. Biemans et al.^114^ calculated the contribution of snow and glacier melt up to 60-70% to total runoff in the UIB. The accumulation and gradual melting of snow is providing a consistent flow to downstream people for hydroelectricity and irrigation in the UIB. Alterations in the snowfall patterns and glacier retreat due to expected warming could significantly affect water availability in future.

The most significant challenge was the underestimation of glacier runoff relying only on discharge data for model calibration. Initially, the GSM-SOCONT model was calibrated using discharge data, yielding a degree-day factor of approximately 0.5 mm/°C/day. This calibration resulted in a glacier melt contribution that was significantly lower than the values reported in the literature in the UIB^117,118^. This led to a discrepancy between observed and simulated glacier runoff, indicating that discharge-based calibration alone failed to accurately account for glacier contributions. According to^17^, modelers can obtain seemingly good calibration results by adjusting the parameters related to snow and ice accumulation and melt processes, although these adjustments may sometimes be based on incorrect assumptions. To resolve this issue, manual calibration was performed using glacier runoff data from the PyGEM model, which resulted in an adjusted degree-day factor of 10.6 mm/°C/day. This adjustment brought the simulated glacier runoff in closer alignment with observed values. It highlights the critical need to integrate additional data sources when calibrating hydrological models in glacierized catchments.

Hydrological models often lack glacier representation, leading to inaccurate runoff estimates^119^. Incorporating glacier mass balance observations into hydrological models, along with limited runoff data, enhances internal consistency, reduces uncertainties^120^, and can enhance the capability of model to predict streamflow in glacier-fed catchments^121^. The adjustment of the glacier degree-day coefficient, based on PyGEM data, significantly improved model performance regarding glacier runoff (Fig. 5). These findings highlight that traditional calibration relying solely on discharge data at the outlet may underestimate glacier contributions in glacierized basin. Previous studies^34,122^ also stresses the importance of accurately parameterizing glacier melt to avoid significant biases in runoff predictions. In this study, integration of glacier runoff data from PyGEM to parameterize the GSM-SOCONT model demonstrates substantial benefits, especially in the highly glaciated UIB, emphasizing the critical role of glacier runoff for accurate hydrological modelling. This is particularly important when seeking to characterise behaviour in remote and extreme altitude study environments e.g. with extensive uninhabitable elevations above 6000 m.

Uncertainties in input data, model parameters, and model structures can have a significant impact on hydrological modelling performance^123^. Precipitation is a major source of uncertainty in hydrological modelling studies in the UIB in simulating runoff components^124–126^. For instance, precipitation estimates in the UIB vary widely ranging from 312 mm according to TRMM satellite data to 1,097 mm from MERRA2 between 1990 and 2011, with ERA5-Land providing an intermediate estimate of 754 mm^127^. We used meteorological data from ERA5 in the GSM-SOCONT model which showed good calibration (NSE = 0.84) and validation (NSE = 0.75) results. The KGE and R metrics followed a similar trend, confirming that the model was well-tuned and captured the hydrological dynamics of the UIB. The observed decrease in performance during validation is consistent with aspects of overfitting^105^. The performance of the GSM-SOCONT model aligns with previous studies using temperature-index model in simulating runoff in glacierized watersheds^128–130^. To improve streamflow predictions, especially for high flows, a hybrid approach combining machine learning and deep learning was employed, using output features from the GSM-SOCONT model as input features for these models.

In the first scenario, three machine learning models like XGBoost, Random Forest Regressor (RFR), and CNN-LSTM were evaluated using meteorological inputs. The stand-alone machine learning models XGBoost and RFR were unable to effectively capture the relationship between temperature, precipitation, evapotranspiration, and runoff. This limitation may be due to the traditional machine learning inability to account for the long-term dependencies in the data required for accurate runoff forecasting^131^. Consequently, predicting runoff using only meteorological features in traditional machine learning models can be challenging. Among the machine learning models, CNN-LSTM demonstrated the best overall performance, particularly during validation (NSE = 0.82). The architecture of the model, which combines convolutional layers for feature extraction with LSTM layers for temporal dependencies, proved effective in capturing both spatial and temporal variability. However, the scatter plots reveal that the model consistently underestimates peak flows, which could compromise its effectiveness in flood risk assessment. The prediction accuracy of timing and magnitude of peak flows is critical during flood management, as these factors are central to assessing and mitigating flood risk.

In this study, CNN-LSTM achieved the best balance between calibration and validation accuracy and selected as a well-suited hybrid model for hydrological predictions. Previous studies also recognized the best performance of CNN-LSTM in hydrological predictions for its ability to effectively capture spatial and temporal patterns in long-term data^132–134^. While CNN-LSTM outperforms other machine learning models in accuracy and robustness, it also shows relatively higher RMSE and MAE compared to other models. However, if a more balanced approach with moderate errors and reasonable generalization is preferred, RFR could be more appropriate. These results suggest that further refinement, such as hyperparameter tuning or incorporating additional features, could enhance the generalization capabilities of models like XGBoost and RFR. The better performance of CNN-LSTM model is consistent with previous studies where deep learning models have proven superior in handling the non-linearity and non-stationarity of hydrological processes, offering better predictive accuracy^58,59,135^.

In scenario 2, incorporation of the GSM-SOCONT model outputs into CNN-LSTM models systematically improved the predictive accuracy of the hybrid models. This study examined various hybrid modelling scenarios by combining meteorological, snow, glacier, and hydrological output features. Among the hybrid models using only meteorological data, the CNN-LSTM3 model showed the best performance during validation. This suggests that additional features could enhance model performance, providing better context for streamflow prediction. Similarly, hybrid models incorporating only snow and glacier output features, such as CNN-LSTM7 and CNN-LSTM14 respectively, demonstrated slight improvement in accuracy than standalone CNN-LSTM model. The inclusion of features like snow water equivalent, glacial series, and total glacier melt provided a more comprehensive representation of the hydrological processes in the UIB. Previous studies also shown that snow and glacier melt components enhance the streamflow predictions in highly glacierized catchment^42,100^. These findings highlight the importance of integrating snow and glacier dynamics, particularly in glacierized catchments where traditional hydrological models may overlook these critical components.

In scenario 3, the use of permutation feature selection and wavelet decomposition techniques further refined the performance of the hybrid models. The use of permutation feature selection results in identifying the most influential features for streamflow prediction, while wavelet transformation allowed the models to capture multiscale patterns and trends in the selected input features, thereby enhancing prediction accuracy of CNN-LSTM19 (NSE = 0.96). The hybrid wavelet data-driven model (CNN-LSTM19) proved to be the best model using valuable information hidden within the time series data. The scatter plots revealed that the hybrid model consistently and accurately predicted peak flows (Fig. 14). The prediction accuracy of timing and magnitude of peak flows is critical during flood management, as these factors are central to assessing and mitigating flood risk.

Previous studies showed that wavelet theory-coupled machine learning models provide better predictions of streamflow than standalone models^136–138^. These findings highlight the importance of hybrid modelling approaches in glacierized catchments like the UIB. Data-driven streamflow forecasting involves hybridizing artificial intelligence techniques with decomposition methods to preprocess and further enhance forecasting accuracy^139,140^. The hybridization of hydrological modelling and machine learning in glacierized catchments offers significant advantages in predictive accuracy and providing a powerful tool for managing water resources in these vulnerable regions. Overall, the integration of physically based model output features with machine learning techniques provided more robust and reliable streamflow predictions. The ability of these hybrid models to capture complex interactions between meteorological, snow, glacier, and hydrological processes sets them apart from standalone approaches.

## Conclusions

Glaciers play a vital role in sustainable water supply downstream, making hydrological modelling essential for predicting future water availability in highly glacierized regions. However, hydrological modelling in these areas is challenging with traditional glacio-hydrological models that have been widely used. In the data-scarce UIB, integration of remote sensing, modelled, and reanalysis products (e.g., ERA5, PyGEM) were helpful for achieving accurate simulation of key hydrological processes like snowmelt and glacier melt. Additionally, advancements in data-driven machine learning models have shown promising results in this data scarce catchment. This study sought to enhance prediction of snow and glacier melt processes and demonstrated that hybrid models, e.g., combining GSM-SOCONT outputs with advanced techniques like wavelet transformation and CNN-LSTM networks, can improve streamflow prediction accuracy. The conclusion about the generalisability of whether data-driven machine learning models can outperform or replace process-based models remains a work in progress, but the major step forwards obtained here is clear.

The calibration of GSM-SOCONT model yielded satisfactory results, with NSE = 0.84, *R* = 0.93, and KGE = 0.85 during the calibration period, slightly reduced in the validation period (NSE = 0.75, *R* = 0.92 and KGE = 0.75). Although the model effectively captures peak flows, it still exhibits significant prediction errors, as indicated by high RMSE and MAE values. The comparison of observed versus simulated glacier runoff revealed underestimation during initial calibration, then model parameters were adjusted using PyGEM data, resulting in a revised glacier melt factor. These results highlight the need to incorporate glacier runoff data for accurate hydrological modelling, especially in glacierized basins like the UIB.

A series of hybrid models were developed by integrating GSM-SOCONT outputs with machine learning models (CNN-LSTM, XGBoost, and RFR). Among these, the CNN-LSTM hybrid model consistently outperformed the standalone models like XGBoost, RFR and GSM-SOCONT in the validation phase, achieving NSE = 0.82 and KGE = 0.88. XGBoost, while highly accurate during training, showed overfitting issues, with a significant drop in performance during validation. Further analysis explored the predictive capability of CNN-LSTM hybrid models using different combinations of inputs derived from GSM-SOCONT model. The performance of best hybrid model for each group of data varied like CNN-LSTM3, CNN-LSTM7, CNN-LSTM10 and CNN-LSTM15 using meteorological input features, and hydrological, snow and glacier output features for GSM-SOCONT model respectively. Among the hybrid models, those incorporating glacier output features (CNN-LSTM11 to CNN-LSTM15) showed outstanding performance during calibration. However, CNN-LSTM14 proved to be the most reliable for predictions, achieving the highest validation NSE (0.83), R (0.91), and the lowest RMSE (892). These results emphasize the critical role of accurately simulating snow and glacier melt components for achieving precise runoff predictions in complex basins like the UIB.

The performance of hybrid models CNN-LSTM1 to CNN-LSTM19 shows progressive improvement in predicting low flow volume (FLV) and high flow volume (FHV). Early hybrid models struggled with large deviations, with FLV errors exceeding 70% and FHV errors around − 30%. However, later hybrid models (CNN-LSTM16, CNN-LSTM17, CNN-LSTM18 and CNN-LSTM19) developed by incorporating permutation feature importance and wavelet transformation techniques enhanced the streamflow prediction. These hybrid models (CNN-LSTM17- CNN-LSTM19) exhibited significant improvements based on FLV and FHV. CNN-LSTM19 achieved the best balance, with FLV overestimated by just 3.53% and FHV underestimated by 2.14%. This trend highlights the gradual refinement of the models, with the later iterations demonstrating superior accuracy and reduced errors in streamflow prediction.

The permutation feature importance technique was crucial in identifying key hydrological, meteorological, and glacial features, leading to the construction of the most effective model, CNN-LSTM16, which showed improved performance over earlier versions. Wavelet transformation was applied to further refine the prediction, with the CNN-LSTM19 model demonstrating the best results. By decomposing time series data into various frequency components, the model effectively captured both low- and high-frequency patterns, leading to significant gains in predictive accuracy. CNN-LSTM19 achieved near-perfect scores during calibration (NSE = 0.99) and maintained high accuracy during validation (NSE = 0.96), indicating strong predictive capabilities. Cross-validation results also confirmed the robustness of CNN-LSTM19 model, showing consistent performance across different folds with minimal overfitting. Overall, this study highlights the importance of hybrid modelling approach by integrating glacio-hydrological simulations, permutation feature importance and wavelet decomposition techniques in enhancing the streamflow simulations, offering a reliable tool for predicting discharge in complex hydrological systems.

The study demonstrates that combining physical models and machine learning (ML) methods for flood prediction in high-altitude mountainous areas offers powerful advances in terms of match-up with observed runoff data. However, issues do remain with interpretability, as ML methods especially deep learning techniques often function as black box, making it difficult to achieve mechanistic understanding of system behaviour. The quality and availability of reliable measurements (spatially and temporally) also poses a significant challenge during validation, their reliance on numerous input features could make them sensitive to data availability and quality.

## Limitations of the study

Combining physical models and machine learning (ML) methods for flood prediction in high-altitude mountainous areas shows promise but challenges remain. The GSM-SOCONT model employs a degree-day approach, which assumes a linear relationship between temperature and glacier melt. This simplification can limit its accuracy in capturing complex glacier melt processes influenced by varying snow cover, albedo changes, or debris layers on glaciers. In data sparse or of low-quality regions, the parameterization may not accurately represent local hydrological processes, leading to potential biases. It can significantly degrade model performance, particularly in high-altitude and remote glacierized basins where monitoring networks are limited.

Integrating physical models and ML methods (hybrid models) can enhance flood prediction accuracy in data-scarce regions. However, lack interpretability especially deep learning techniques often function as black boxes, making it difficult to understand the factors contributing to streamflow fluctuations. Hybrid models can obscure the distinct contributions of glacier melt, snowmelt, and other factors, making it difficult to achieve precise simulations and informed decision-making. In the proposed framework, the accuracy and generalizability of the hybrid models are heavily relying on parameterization, meaning how well its key settings (parameters) are chosen and fine-tuned. Furthermore, integrating complex models with multiple parameters can increase the risk of overfitting, where the model performs well on the training data but poorly on unseen data, as seen with the best-performing models (RFR and XGBoost).

Future research should explore the integration of additional data sources, such as remote sensing products, to further enhance model accuracy. Moreover, expanding the study to include other glacierized catchments with different climatic conditions could validate the generalizability of the proposed hybrid approach. For future improvements, the study recommends exploring ensemble modelling techniques, which integrate various modelling approaches (e.g., conceptual models, machine learning, decomposition methods) to enhance prediction accuracy by mitigating uncertainties associated with individual models. Additionally, incorporating advanced uncertainty analysis methods, like Monte Carlo simulations, is suggested to better quantify and manage uncertainties in predictions, offering decision-makers a clearer range of possible outcomes. Lastly, extending the validation period to include long-term temporal data, covering multiple years or decades, is recommended to assess the robustness of operational models acknowledging and accommodating non-stationarity associated with atmospheric and oceanic circulation and anthropogenic climate change. Despite these challenges, integrating physical models and ML methods can enhance flood prediction accuracy in data-scarce regions.

## Electronic supplementary material

Below is the link to the electronic supplementary material.

Supplementary Material 1

## Data Availability

The datasets used and/or analysed during the current study available from the corresponding author on request.
