# Supplementary material for: Enhanced streamflow forecasting using hybrid modelling integrating glacio-hydrological outputs, deep learning and wavelet transformation
Source: Sci Rep. 2025 Jan 22;15:2762. doi: 10.1038/s41598-025-87187-1 (PMC11754805; doi:10.1038/s41598-025-87187-1)
Supplement: Supplementary file 1 — Supplementary Material 1 [file 41598_2025_87187_MOESM1_ESM.pdf]

Enhanced Streamflow Forecasting Using Hybrid Modelling Integrating Glacio-Hydrological Outputs, Deep Learning and Wavelet Transformation

Jamal Hassan Ougahi[1,2,3] and John S Rowan[1]

1. *UNESCO Centre of Water Law, Policy & Science, University of Dundee, UK*
2. *Higher Education Department, Government of the Punjab, PK*
3. *Corresponding author*

Table 1 Meteorological Combinations, Including Temperature (T), Precipitation (P), and Potential Evapotranspiration (PET), as Inputs to the CNN-LSTM Models.

Models	Metrics	CNN-LSTM1	CNN-LSTM2	CNN-LSTM3
Calibration	NSE	0.67	0.79	0.80
	KGE	0.70	0.74	0.73
	R	0.82	0.89	0.89
	RMSE	1413	1127	1115
	MAE	747	614	609
Validation	NSE	0.70	0.77	0.82
	KGE	0.81	0.87	0.89
	R^2	0.84	0.88	0.91
	RMSE	1180	1032	917
	MAE	736	573	526

Table 2. Snow outputs from the GSM-SOCONT model, including snowmelt discharge (Qsnow), snow water equivalent for the basin (SWEbasin), snow series data (Sseries), and precipitation equivalent for the basin (Peqbasin), as inputs to the CNN-LSTM model.

Models	Metrics	CNN-LSTM4	CNN-LSTM5	CNN-LSTM6	CNN-LSTM7
Calibration	NSE	0.77	0.86	0.89	0.90
	KGE	0.83	0.88	0.86	0.87
	R	0.88	0.93	0.95	0.95
	RMSE	1195	926	809	797
	MAE	790	530	431	427
Validation	NSE	0.68	0.84	0.82	0.82
	KGE	0.76	0.85	0.90	0.90
	R^2	0.83	0.91	0.91	0.91
	RMSE	1220	877	921	918
	MAE	768	480	521	520

Table 3. Hydrological outputs from the GSM-SOCONT model, including baseflow (BF), evapotranspiration (ET), and surface runoff (SR), used as inputs to the CNN-LSTM model.

Models	Metrics	CNN-LSTM8	CNN-LSTM9	CNN-LSTM10
Calibration	NSE	0.79	0.84	0.86
	KGE	0.86	0.81	0.84
	R	0.89	0.91	0.93
	RMSE	1135	1001	916
	MAE	799	574	515
Validation	NSE	0.76	0.81	0.81
	KGE	0.81	0.90	0.90
	R	0.87	0.90	0.90
	RMSE	1069	936	949
	MAE	715	537	528

Table 4 Glacial outputs from the GSM-SOCONT model, including total glacial melt discharge (Qgl_tot), glacial series (Gseries), snow water equivalent for glaciers (SWEgl), precipitation equivalent snowmelt for glaciers (PeqGl), and glacial melt (Qgl), used as inputs to the CNN-LSTM model.

Models	Metrics	CNN-LSTM11	CNN-LSTM12	CNN-LSTM13	CNN-LSTM14	CNN-LSTM15
Calibration	NSE	0.53	0.77	0.80	0.86	0.87
	KGE	0.58	0.72	0.77	0.80	0.83
	R	0.73	0.88	0.89	0.93	0.93
	RMSE	1692	1179	1105	910	901
	MAE	1107	624	602	503	500
Validation	NSE	0.67	0.81	0.81	0.83	0.81
	KGE	0.77	0.89	0.86	0.88	0.86
	R	0.82	0.90	0.90	0.91	0.90
	RMSE	1248	956	943	892	941
	MAE	922	567	550	544	581

Table 5. Performance evaluation metrics of CNN-LSTM hybrid models based on features selected from the GSM-SOCONT model and decomposed using Symlet, Daubechies (db), and Coiflet (coif) filters.

Models	Metrics	CNN-LSTM17	CNN-LSTM18	CNN-LSTM19
Calibration	NSE	0.99	0.99	1.00
	KGE	0.97	0.98	0.99
	R	1.00	1.00	1.00
	RMSE	226	201	143
	MAE	118	101	69
Validation	NSE	0.82	0.87	0.96
	KGE	0.89	0.90	0.96
	R	0.91	0.93	0.98
	RMSE	910	783	442
	MAE	495	435	229

Table 6. Performance metrics illustrating 5-fold cross validation results for training and test data for CNN-LSTM19 model.

	Folds	RMSE	MAE	NSE	KGE	R
Calibration	k1	381	194	0.98	0.96	0.99
	k2	463	201	0.97	0.95	0.98
	k3	439	208	0.97	0.96	0.98
	k4	336	177	0.98	0.99	0.99
	k5	333	174	0.98	0.99	0.99
Validation	k1	399	216	0.97	0.96	0.98
	k2	454	224	0.96	0.96	0.98
	k3	362	191	0.97	0.98	0.99
	k4	439	220	0.96	0.93	0.98
	k5	372	187	0.97	0.98	0.98
